# Methodological problems of SARS-CoV-2 rapid point-of-care tests when used in mass testing

**DOI:** 10.3934/publichealth.2022007

**Published:** 2021-11-23

**Authors:** Oliver Hirsch, Werner Bergholz, Kai Kisielinski, Paul Giboni, Andreas Sönnichsen

**Affiliations:** 1 Department of Psychology, FOM University of Applied Sciences, Birlenbacher Str. 17, 57078 Siegen, Germany; 2 International Standards Consulting GmbH, 30989 Gehrden, Germany; 3 Private Practice, 40212 Düsseldorf, Germany; 4 Private Practice, 22763 Hamburg, Germany; 5 Department of General Practice and Family Medicine, Center for Public Health, Medical University of Vienna, Wien, Austria

**Keywords:** SARS-CoV-2, COVID-19, point-of-care diagnostics, rapid testing, methodology

## Abstract

The aim of the current study is to perform model calculations on the possible use of SARS-CoV-2-rapid point-of-care tests as mass tests, using the quality criteria extracted from evidence-based research as an example for the Federal Republic of Germany. In addition to illustrating the problem of false positive test results, these calculations are used to examine their possible influence on the 7-day incidence. For a substantial period of time, this parameter formed the decisive basis for decisions on measures to protect the population in the wake of the COVID pandemic, which were taken by the government. Primarily, model calculations were performed for a base model of 1,000,000 SARS-CoV-2-rapid point-of-care tests per week using various sensitivities and specificities reported in the literature, followed by sequential testing of the test positives obtained by a SARS-CoV-2 PCR test. Furthermore, a calculation was performed for an actual maximum model based on self-test contingents by the German Federal Ministry of Health. Assuming a number of 1,000,000 tests per week at a prevalence of 0.5%, a high number of false positive test results, a low positive predictive value, a high negative predictive value, and an increase in the 7-day incidence due to the additional antigen rapid tests of approx. 5/100,000 were obtained. A previous maximum calculation based on contingent numbers for self-tests given by the German Federal Ministry of Health even showed an additional possible influence on the 7-day incidence of 84.6/100,000. The model calculations refer in each case to representative population samples that would have to be drawn if the successive results were comparable which should be given, as far-reaching actions were based on this parameter. The additionally performed SARS-CoV-2-rapid point-of-care tests increase the 7-day incidence in a clear way depending on the number of tests and clearly show their dependence on the respective number of tests. SARS-CoV-2-rapid point-of-care tests as well as the SARS-CoV-2-PCR test method should both be used exclusively in the presence of corresponding respiratory symptoms and not in symptom-free persons.

## 1. Introduction

SARS-CoV-2 rapid tests have attracted attention as a supplement or alternative to SARS-CoV-2 PCR testing because of the possibility of rapid on-site testing (“point of care test”, POCT) and have been included in the national testing strategy of the Federal Ministry of Health of the Federal Republic of Germany dated 2020/10/14 as well as of individual federal states. Rapid antigen tests are medical devices that can currently be certified by the manufacturers themselves and labelled with a CE label (Conformité Européenne). Not only in Germany, but in many other countries, rapid SARS-CoV-2 antigen testing is considered a promising method for containing and combatting the SARS-CoV2 pandemic. Since the POCT tests are to be used as mass tests in certain occupational sectors, e.g. in schools or in a day-to-day setting for entering restaurants and shops, it is necessary to take a closer look at them from an evidence-based and test-theoretical point of view.

In this context, it is interesting to take a closer look at the original instructions for the use of rapid antigen tests. Such instructions are available for the SARS-CoV-2 Rapid Antigen Test of the company SD Biosensor from South Korea, distributed by Roche. First of all, it is pointed out that gloves and lab coats are to be worn when handling the reagents. The samples and materials used must be disposed of as biohazardous waste. Therefore, a simple disposal via household waste seems questionable. The test result should be read after 15–30 minutes. If the test result is read after more than 30 minutes, the test result could be wrong. The test result should not be used as the sole basis for a decision. Further package inserts are available from the MEDsan and Hotgen tests, which essentially confirm the above information.

The position paper of the nationwide research network Applied Surveillance and Testing in the National Research Network of University Medicine on COVID-19 on the application and approval practice of rapid antigen tests for the detection of the new coronavirus, SARS-CoV-2, contains some findings and recommendations in this matter [1]. With correct swab collection and test performance, highly infectious individuals could be rapidly identified. The diagnostic sensitivity of the rapid antigen tests is limited. Despite having a negative result, the appropriate hygienic measures must continue to be observed. It is demanded that only rapid antigen tests should be used that have fulfilled the minimum criteria (sensitivity >80%, specificity >97%) required by the German Paul Ehrlich Institute (PEI), the WHO and the European Centre for Disease Prevention and Control (ECDC) in independent, published validation studies [2]. These values are also confirmed by an explanatory paper of the German Robert Koch Institute [3]. Rapid antigen tests should be carried out and evaluated under the supervision of trained medical personnel. The validity of the result is limited to about 24 hours. In a group of asymptomatic persons, the number of false-positive results in rapid antigen tests may exceed the number of true-positive results. This is a known phenomenon when the occurrence of the disease is rare (low prevalence). The validation of the tests should be done in controlled studies by independent scientists. In future the approval of such diagnostic medical devices should be based on a manufacturer-independent validation process.

A Cochrane review looked at rapid and molecular-based SARS-CoV-2 antigen tests. Publications up to mid-November 2020 were screened [4]. A total of 64 studies were included, 20 of which were preprints without peer review, examining 16 antigen tests and 5 molecular tests. Numerous sources of bias were found in the studies, e.g. in the selection of participants, the lack of definition of a reference standard for the absence of infection and representation of the participant flow. The antigen tests showed considerable differences in sensitivity between the studies. Sensitivity was high in studies with low PCR cycle threshold (Ct) <25 (94.5%, 95% CI 91.0% to 96.7%) compared with Ct values >25 (40.7%, 95% CI 31.8% to 50.3%). The overall specificity was 99.6% (95% CI 99.0% to 99.8%). For molecular tests, sensitivities ranged from 73.0% to 100% and specificities from 97.2% to 99.7%. However, these average values are not meaningful for the use of the tests in a specific setting and should therefore not be used globally. The authors conclude that the rapid antigen tests can detect individuals with high viral loads. Data on asymptomatic individuals are limited. In these, rapid antigen tests had an average sensitivity of 58.1% (95% CI 40.2% to 74.1%) and an average specificity of 98.9% (95% CI 93.6% to 99.8%). They also criticize that there is no reference standard for contagiousness. The RT-PCR method still detects viral RNA weeks after an infection and thus falsely classifies people as infectious. The classification of Ct values into infectious/non-infectious categories is problematic because the correlation between Ct values and viral load varies between different devices and laboratories. Thus, a gold standard against which the rapid tests could be validated is obviously missing, although the studies claim that the SARS-CoV-2 PCR test is this gold standard [4] (p 3). Furthermore, with a low prevalence of the disease, a significant number of false positive test results are to be expected. According to the authors more evidence is needed for the use of rapid tests in asymptomatic persons. In repeated testing—where the entire testing strategy would then have to be examined and not just the tests used—and in testing outside the health care setting such as schools, and in self-testing in general, as in most studies, this was carried out by professionals. It is also criticized that the test manufacturers' instructions were often not adhered to, e.g. frozen samples were used or a longer time elapsed between sampling and testing. Due to the relatively high number of false-positive tests with a low prevalence among asymptomatic persons, a correspondingly high number of follow-up RT-PCR tests are required to prevent unnecessary quarantine measures. The effectiveness of mass testing can only be investigated by cluster-randomized community trials [4] (p 40).

A previous combined systematic review and meta-analysis of ten studies yielded an overall sensitivity of 64.8% and a specificity of 98.0% with high heterogeneity between studies [5]. The authors interpret the low sensitivity compared to SARS-CoV-2 PCR testing to imply that asymptomatic test-positive individuals would not be detected with rapid antigen tests and that they are therefore unsuitable for mass testing. The extent to which this conclusion is correct will be discussed later.

We conducted an extensive literature search in the standard databases to find studies that went beyond the Cochrane Review by Dinnes et al. [4] and the review and meta-analysis by Riccò et al. [5]. The ECDC Technical Report contains further studies that have examined rapid antigen tests [2]. When considering the quality criteria listed in Annex 1 of this publication, it can be assumed that the studies discussed here represent an appropriate cross-section.

The test of the company SD Biosensor was examined in the study by Paul et al. with a cross-sectional design [6]. However, a fundamental problem already becomes clear here as the authors state that the previous gold standard for the detection of a SARS-CoV-2 infection is real-time PCR (RT-PCR) and this in turn is used to validate the rapid antigen test in this and other studies. From a test theoretical point of view this represents a serious substantial problem. After all, PCR testing in the field of SARS-CoV-2 has not been standardized or validated and cannot directly detect a virus capable of replication [7]. For its part, the SARS-CoV-2 PCR test method [8] was tested for its quality criteria in a round robin interlaboratory comparison and a sensitivity of up to 99.7% and a specificity of up to 98.6% can be assumed [9]. In the study by Paul et al., testing was carried out by trained health care professionals and nurses in an interdisciplinary emergency department. The sensitivity in the total sample was 71.7%, the specificity 99.5%. In asymptomatic but PCR-test-positive patients the sensitivity was as low as 38.9% and the specificity 99.7%; in persons with symptoms the sensitivity was 85.7%, the specificity 98.3% [6]. The study also shows that the Ct value is apparently the parameter with the largest impact on the result in SARS-CoV-2 PCR testing. From a Ct value of 22 and smaller, the detection rate of the rapid antigen test was 100%. Consequently, the rapid antigen test is particularly reliable in detecting those individuals with a high viral load. However, these came exclusively from symptomatic persons. These results were confirmed in another study by Scohy et al. which reasonably demonstrates that sensitivities of SARS-CoV-2 rapid Point-of-Care tests drop substantially if low viral loads were present as Ct values above 30 [10].

At two universities in Wisconsin, USA, n = 871 asymptomatic and n = 227 symptomatic individuals were tested with the Sofia SARS Antigen Fluorescent Immunoassay (FIA) and these results were associated with one RT-PCR test each [11]. Students and staff were offered testing and samples were collected by health workers in one university and by the participants themselves under supervision in the other university. In the group of asymptomatic persons, 2.0% were positive in the RT-PCR test. This resulted in a sensitivity of 41.2% and a specificity of 98.4%. In the group of symptomatic persons, 17.6% were positive in the RT-PCR test. This resulted in a sensitivity of 80.0% and a specificity of 98.9%. Virus detection by cell culture was successful for 34 (46.6%) out of 73 samples indicated as positive by both tests.

Further studies on quality criteria of rapid antigen tests are referenced in Supplementary Material S1 and are referred to here as additional information.

The aim of the current study is to carry out model calculations on the possible use of rapid antigen tests as mass tests using the quality criteria which could be extracted from evidence-based research, e.g. for the Federal Republic of Germany. These calculations should also be used to examine their possible impact on the 7-day incidence since this parameter represented the basis for decision making by the federal and state governments for measures in the wake of the COVID-19 pandemic for a substantial period of time.

Since September 2021, the nationwide incidence has been replaced by the hospitalization rate as a primary metric for the pandemic due to a change in the Infection Protection Act, although the 7-day incidence is still highly present in the media and politics, which means that this parameter is still important and relevant. The hospitalization rate provides information about the burden in hospitals. The value indicates how many patients infected with Covid-19 are hospitalized per 100,000 inhabitants within seven days. Thresholds for corresponding measures were set at 3, 6, and 9 patients per 100,000 inhabitants.

## 2. Materials and methods

The 7-day incidence counts the absolute number of new cases that occurred within 7 days in a certain geographical region. In the Federal Republic of Germany this is currently standardized to a population of 100,000.

At this point, it should only be pointed out that the 7-day incidence based on positive SARS-CoV-2 PCR tests does not represent a valid epidemiological parameter, as it is to be regarded as dependent on the number of tests involved and the details of the preselection of the persons tested. On the basis of the 7-day incidence it must be noted that it is unclear where the tests come from, i.e. which testing strategy was pursued. The primary testing of persons with symptoms yields a higher test positive rate than the primary testing of persons without symptoms, whereby especially the latter is associated with a high risk of false positive results. Studies of representative population samples would provide more clarity here, but they are not carried out in this format.

According to the Robert Koch Institute (RKI), only positive rapid antigen tests that were subsequently confirmed by a positive SARS-CoV-2 PCR test are included in the official calculations [12]. However, only 65% of the positive rapid antigen tests were subsequently examined by a PCR test and no evidence can be provided about the completeness of the positive antigen detections reported to the health authorities.

The prevalence of COVID-19 cannot be reliably stated for Germany as no epidemiological studies of representative samples are available. Estimates referring to the year 2020 range between 0.4 and 1.8% [13]. Katharina Schüller, head of the German Statistical Society, assumes that a prevalence estimate of currently 0.5% is appropriate for Germany given that the Robert Koch Institute estimates the number of unreported cases by a factor of 5 [14]. This value is confirmed by studies from England with prevalences depending on the season of 0.4–1% [15], 0.15% in Slovenia [16], 0.6% in Iceland [17], 0.3% in Luxembourg [18]. Therefore, a prevalence estimate of 0.5% will be used for the calculation examples for the sequential testing strategy seeing that this value seems reasonable for a point prevalence of this disease in spring/summer. Sequential means that in everyday life a rapid antigen test is carried out first. If this shows a positive result, the respective person must undergo a SARS-CoV-2 PCR test. Furthermore, the calculation examples are also carried out using parallel test strategies specified in the respective studies (simultaneous performance of rapid antigen tests and PCR tests) and the original prevalences.

According to the management report of the Robert Koch Institute, at least 1.1 million SARS-CoV-2 PCR tests have been carried out consistently every week since the beginning of 2021 and as many as 1.4 million in week 12 according to the daily status report of 31.3.2021 [19].

The calculations, the results of which are reported below, were each carried out for 1,000,000 rapid tests per week. This appears to be an absolute lower limit in view of the free availability for these tests in discounters such as ALDI and LIDL and the compulsory testing in schools. No concrete sales or implementation figures for the tests could be ascertained so far or they have not been made public. Furthermore, in view of the similarly performed number of SARS-CoV-2 PCR tests per week this presumed number seems appropriate as a basic model. In the meantime, actual test numbers can be multiplicatively adjusted to these models.

On the website of the Federal Ministry of Health it can be noted that for the months of March and April 2021 call-off quotas amounting to 132.5 million self-tests have been made available to the federal states [20]. This would correspond to a weekly maximum of 132.5 million tests/8 weeks = approx. 16.5 million tests/week. A model calculation should therefore also be made for this case.

The population of the Federal Republic of Germany on 2020/09/30 was 83,190,556 [21]. This population level is used as the basis for standardizing the calculated positive test results to a 7-day incidence per 100,000 inhabitants which was declared by the German government. It is calculated as the total number of positive tests in the past 7 days divided by 83,190,556 and multiplied by 100,000. A simplified calculation is: total number of positive tests in the past 7 days divided by 831.9.

The calculations shown were carried out using the Diagnostic test calculator by Alan Schwartz, University of Illinois, Chicago (http://araw.mede.uic.edu/cgi-bin/testcalc.pl). Furthermore, the R package mada, a tool for the meta-analysis of diagnostic accuracy, was used. R version 4.02 and RStudio 1.2.5042 were used.

The following parameters are calculated by the mentioned programmes:

The sensitivity of a test is the proportion of patients with the disease for whom the test is positive for the disease. It is calculated as the quotient of the true positives (TP) divided by the sum of the true positives (TP) plus the false negatives (FN): TP/(TP + FN).

The specificity of a test is the proportion of patients without the disease for whom the test is negative for the disease. A specific test is thus unlikely to classify people as having the disease if they do not have it. It is calculated as the quotient of the true negatives (TN) divided by the sum of the true negatives (TN) plus the false positives (FP): TN/(TN + FP).

The positive predictive value (PPV) indicates the proportion of people who actually have the disease out of all those with a positive test result. It is calculated as the quotient of the true positives (TP) divided by the sum of the true positives (TP) plus the false positives (FP): TP/(TP + FP).

The negative predictive value (NPV) indicates the proportion of people who are actually healthy out of all those with a negative test result. It is calculated as the quotient of the true negatives (TN) divided by the sum of the true negatives (TN) plus the false negatives (FN): TN/(TN + FN) [22].

## 3. Results

In the following, simulation calculations will be carried out with some selected study data from the introduction under the conditions explained in the method section.

**Figure 1.**
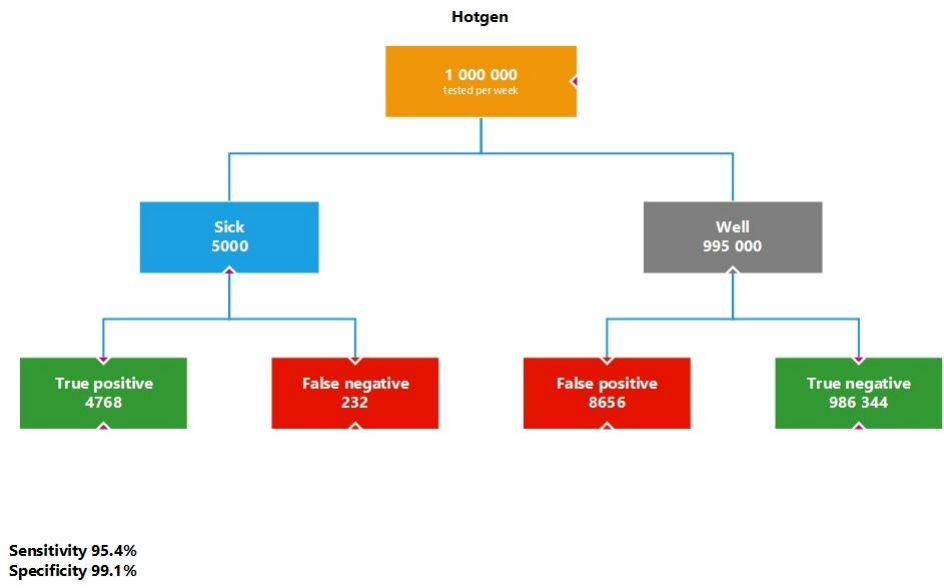
Simulation calculation for the Hotgen antigen rapid test with sequential testing strategy and prevalence of 0.5%.

First of all, the quality criteria of the Hotgen test stated by the manufacturer (sensitivity 95.37%, specificity 99.13%, see above) are to be applied to a sequential test design, which takes place for example in a mass test. If we transfer the values of the Hotgen antigen rapid test to such a scenario and use the prevalence of 0.5% for Germany explained in the methods section, we obtain 8656 false positives from 1 million measurements (Figure 1). The positive predictive value (PPV) is 35.52%, which means that 35.52% of the persons who show a positive test result are actually positive for SARS-CoV-2. The negative predictive value (NPV) is 99.98%, which means that 99.98% of those who test negative are actually considered negative for SARS-CoV-2.

**Figure 2.**
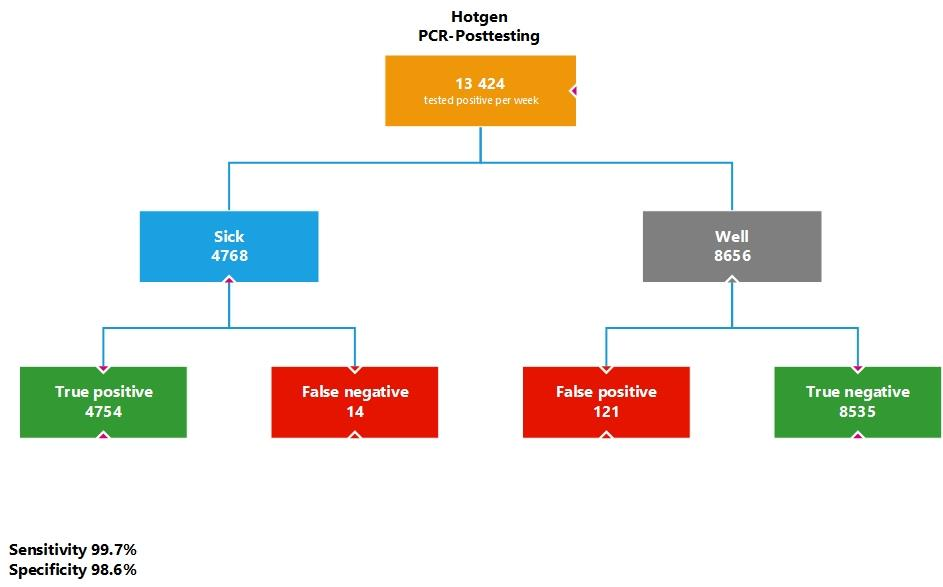
Simulation calculation for the PCR post-testing of the positive test results of the Hotgen antigen rapid test from Figure 1 with a pre-test probability of 35.52%.

If the 13,424 people identified as positive (4768 true positives + 8656 false positives) are now retested with a SARS-CoV-2 PCR test (sensitivity 99.7%, specificity 98.6%), a prevalence of 35.52% is assumed, as this is the pre-test probability to the extent of the PPV. Under these assumptions, there would still be 121 individuals as false positives who would be wrongly sent to quarantine. The additional rapid antigene tests would increase the 7-day incidence by (4754 + 121)/831.9 = 5.86/100,000 (Figure 2).

Table 1 shows the summary of the results of the simulation calculations for the rapid antigen tests for some example studies described in the introduction. The detailed analyses of these studies can be found in Supplementary Material S1.

**Table 1.** Results of the simulation calculations of rapid antigen tests with the data from the respective studies described in the introduction. These were performed for 1,000,000 tests per week and 0.5% prevalence. Only the calculation for the FMH data was carried out with 16.5 million tests.

Study data	True positive	False negative	False positive	True negative	PPV	NPV
Hotgen	4768	232	8656	986,344	35.52	99.98
Cochrane	2905	2095	10,945	984,055	20.98	99.79
Wisconsin	2060	2940	15,920	979,080	11.46	99.70
Robert-Koch-Institute	4000	1000	19,900	975,100	16.74	99.90
German Federal Ministry of Health (FMH)	66,000	16,500	328,350	16,089,150	16.74	99.94

It can be clearly seen that as the number of tests increases, the number of false positives increases significantly. This is reflected in the figures of the German Federal Ministry of Health, which assume a number of 16.5 million tests per 7 days. This illustrates that an increasing absolute number of tests results in an increasing absolute number of false positive test results.

The negative predictive value (NPV) is to be regarded as very high, while the positive predictive value (PPV) is to be classified as low.

The persons with positive test results (true and false positives) are then subjected to SARS-CoV-2 PCR testing in accordance with the sequential testing strategy, the results of which are shown in Table 2.

**Table 2.** Results of the simulation calculations of sequential SARS-CoV-2 PCR tests with the data from the respective studies described in the introduction. These were based on 1,000,000 tests per week and 0.5% prevalence. Only the calculation for the FMH data was carried out with 16.5 million tests.

Study data	True positives after PCR	False negative after PCR	False positives after PCR	True negative after PCR	Increase in incidence per 100,000
Hotgen	4754	14	121	8535	5.86
Cochrane	2897	9	153	10,791	3.67
Wisconsin	2054	6	223	15,697	2.74
Robert-Koch-Institute	3989	12	279	19,621	5.13
German Federal Ministry of Health (FMH)	65,816	198	4597	323,739	84.64

**Figure 3.**
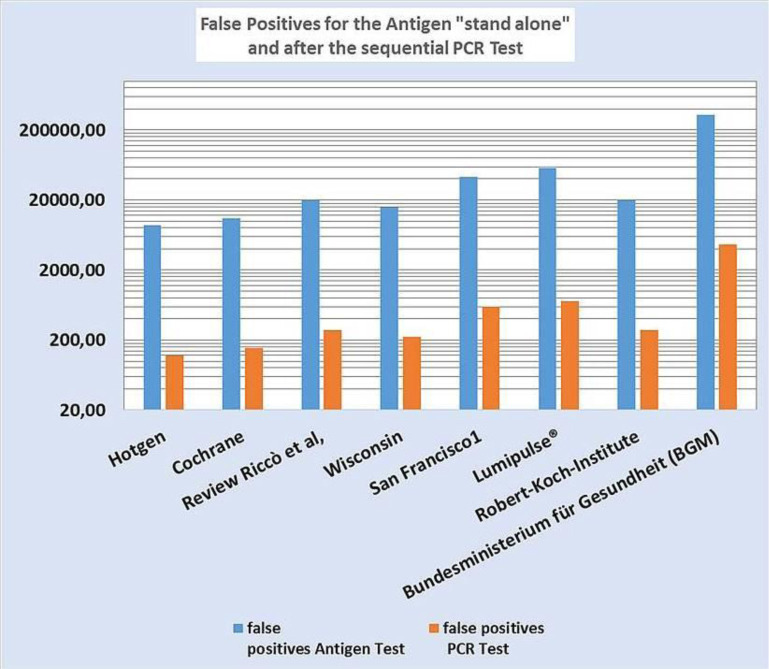
Number of false positive test results after rapid antigen testing and after subsequent SARS-CoV-2 PCR testing with the data from the respective studies in the results section. These were extrapolated to 1,000,000 tests per week and 0.5% prevalence. Only the calculation for the German Federal Ministry of Health (Bundesministerium für Gesundheit, BGM, English abbreviation: FMH) data was carried out with 16.5 million tests. The results related BGM (FMH) can be converted into the figures of the Robert-Koch-Institute by dividing them by 16.5 because they are based on the same statistical quality criteria.

The high number of false positive test results after rapid antigen testing can be significantly reduced by retesting with a SARS-CoV-2 PCR test, however there are still potentially many thousands of people—especially if the maximum values of the German Federal Ministry of Health are assumed—who are wrongfully being quarantined, which has significant psychological, social and economic impacts. This is illustrated once again in Figure 3 which also displays data from studies whose detailed analyses are presented in Supplementary Material S1.

The impact on 7-day incidence of the additional rapid antigen tests and sequential testing by SARS-CoV-2 PCR test is moderate at 1 million rapid antigen tests, but increases massively at higher test numbers, showing the dependence of 7-day incidence on the number of tests. It is assumed here that, for the purpose of comparability, representative population samples are always taken from the general population with a prevalence of, in this case, 0.5 %. If, for example, testing is carried out in low prevalence settings, the influence of the additional tests on the 7-day incidence will be significantly lower (see Supplementary Material S2). The clinical validity of such an incidence increase will be addressed in more detail in the discussion.

**Figure 4.**
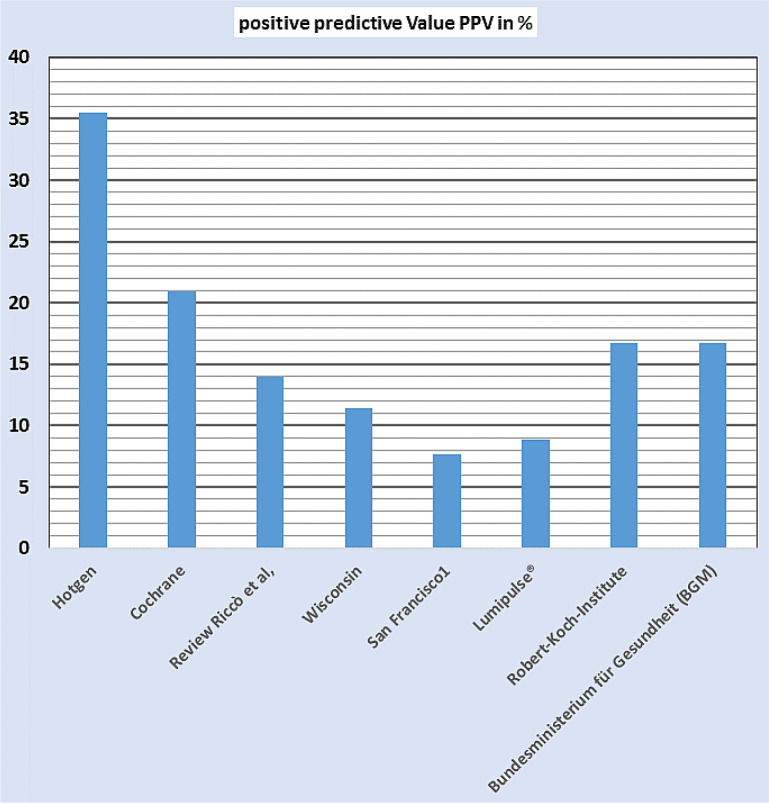
Positive predictive value (PPV) after rapid antigen testing with the data from the respective studies in the results section. These were transferred to 1,000,000 tests per week and 0.5% prevalence. Only the calculation for the German Federal Ministry of Health (Bundesministerium für Gesundheit, BGM, English abbreviation: FMH) data was carried out with 16.5 million tests.

The positive predictive value (PPV), i.e. the proportion of people with a positive test result who are actually diagnosed with COVID-19, must be considered rather low as the majority of values are in the <20% range making the usefulness of such a test strategy questionable (Figure 4). This is also criticized in the current Recommendations for national SARS-CoV-2 testing strategies and diagnostic capacities of the WHO [23].

The negative predictive value (NPV), i.e. the proportion of people with a negative test result who are not actually diagnosed with COVID-19, is high. Rather theoretically, a false negative test could give infected people access to a number of situations which are under restrictions, like airplane flights or gym. One should keep in mind that rapid antigen tests used in mass testing are performed in asymptomatic individuals, while symptomatic persons are forwarded to medical care institutions for further evaluation. However, from an empirical perspective until now, there is no empirical data which would prove the assumption that asymptomatic people would significantly contribute to causing a SARS-CoV-2-infection [23]. On the contrary, there is robust empirical data from a study conducted in Wuhan with 10 million contacts and PCR testing showing that even asymptomatic individuals who tested positive do not cause any infections [24]. Thus, according to these experimental data, an asymptomatic state is not related to infectivity. Hence, the majority of COVID-19 cases are asymptomatic [25], the hypothesis of asymptomatic and presymptomatic transmission is widely supported, however the scientific literature does not provide strong evidence for asymptomatic spread of the disease in the general population [26]. The main arguments for asymptomatic spread are case reports and expert opinions [27]–[31]. Empirically, the assumption that asymptomatic persons are virus spreaders cannot be supported [26]. Systematic reviews do not provide moderate or strong evidence [32]–[34],[26]. Therefore, the WHO does not recommend the general testing of asymptomatic individuals, but only in special environments [23]. The increase in the 7-day incidence due to the additional rapid antigen tests carried out is, according to the simulation calculations, approx. 5/100,000 for 1,000,000 tests. This is to be regarded as a basic value that can serve as a multiplier if the actual number of tests per week is known. How severe the influence of a high number of additional rapid antigen tests with subsequent SARS-CoV-2 PCR testing can be on the incidence (additional 84.6/100,000) is shown by the maximum example based on the contingent figures of 16.5 million tests per week given by the German Federal Ministry of Health (Bundesministerium für Gesundheit, BGM, English abbreviation: FMH).

## 4. Discussion

### 4.1. Relevance of results

The simulation calculations for the Federal Republic of Germany on the basis of the evidence-based literature on SARS-CoV-2 antigen rapid tests yielded the following key results: on the assumption of 1,000,000 tests per week with a prevalence of 0.5%, a high number of false positive test results, a low positive predictive value, a high negative predictive value, but with a significant number of potentially missed cases of infection and an increase in the 7-day incidence due to the additional rapid antigen tests of approx. 5/100,000. A previous maximum calculation based on the contingent numbers for self-tests given by the BGM (FMH) even showed an additional possible influence on the 7-day incidence of 84.6/100,000. These calculations are based on the assumption of representative population samples from the general population with a prevalence of 0.5%.

It could now be argued that the people who were classified as false positive have to be rescreened with a PCR test anyway and that this would then detect these false positive results. This is certainly correct for the majority of those affected as our calculations show although at least several hundred people or even potentially many thousands of people—depending on the absolute number of tests—would still be unjustly sent into isolation, accompanied by psychological and economic harm. In addition, it is mandatory for all those tested positive to go into quarantine immediately after the positive test until the PCR result is available, usually after 1–2 days.

To make matters worse, the Robert Koch Institute reports that only 65% of the positive rapid antigen tests it received were followed up with a PCR test [12]. This is also confirmed by data from South Tyrol (Provincia autonoma di Bolzano – Alto Adige), Italy. Since the rapid antigen tests have been used there is no correlation between the number of rapid antigen tests and the number of PCR tests that can be ascertained (http://www.provinz.bz.it/sicherheit-zivilschutz/zivilschutz/aktuelle-daten-zum-coronavirus.asp#accept-cookies). From the data obtained from South Tyrol it can be further concluded that after testing more than 80% of the population the test positive numbers only temporarily decreased by about 1/3.

In addition, it must be taken into account that a positive antigen rapid test result can set in motion a further, depending on the setting, test cascade of contact persons, which in turn can lead to the complications shown in the example calculations. If there are enough contact people (e.g. in a school class), then an exponential growth through several “generations” of contact people, stimulated by predominantly false positives, can arise.

The group of authors of the Robert Koch Institute also addresses the lower sensitivity and lower specificity of rapid antigen tests and the resulting false negative and false positive results. The false positive results could be at least partially detected with a subsequent PCR test [35]. The sample collection is very decisive for the accuracy of the result as are environmental variables such as temperature. However, medical laypeople cannot effectively control the sample quality. These tests are intended for use by professionals. A positive result does not mean a diagnosis of SARS-CoV-2 infection. The diagnosis is only made by the subsequent RT-PCR test and the medical assessment. This corresponds to the recommendation of the WHO [36] but is not actually implemented in Germany since a positive SARS-CoV-2 PCR test result alone has so far been misinterpreted as an infection without taking into account symptoms or Ct values and therefore does not meet the requirements of the Infection Protection Act.

The authors of the RKI also state that the national testing strategy provides for targeted and occasion-related testing. This is not to be regarded as fulfilled in the case of mass testing of asymptomatic individuals. If a positive rapid antigen test result occurs, then there is an obligation for immediate domestic isolation and retesting by means of RT-PCR screening. Experience with adequately suitable antigen tests would hardly be available in Germany [35]. The clinical evidence-based paradigm of testing for differential diagnostic clarification and verification of a suspected diagnosis made clinically on the basis of symptoms is not observed in this approach.

The false negative test results would fundamentally question the goal of preventing the spread of infection in the long term. On the other hand, it must be considered that the rapid antigen tests correctly respond to people with a low viral load and high SARS-CoV-2 PCR-Ct values by giving negative results; and the definition of a false negative result is due to the dubious gold standard of the PCR test. The pseudo-validation studies carried out clearly show that persons with SARS-CoV-2 PCR test Ct values ≥30 are not likely to be infectious [37],[38], this may be the case even for Ct values >24 [39],[40]. In comparison to symptomatic patients asymptomatic SARS-CoV-2 PCR test positive patients have a low viral load and also only short viral excretion times, which considerably reduces their risk of transmitting SARS-CoV-2 [24]. The clinical significance of a postulated 2.6- and 8-fold more SARS-CoV-2 infections than those identified by testing [41] has to be questioned in view of the non-existent over-load of the German health care system [42] and a non-existent excess mortality in 2020 [43]. A study in a skilled nursing facility came to different conclusions saying that more than half of residents with positive test results were asymptomatic at the time of testing and most likely contributed to transmission [44]. However, the authors were not able to quantify the contributions of asymptomatic and presymptomatic subjects to transmission of SARS-CoV-2. There was a poor correlation between symptom onset and viral shedding as there were difficulties of measuring precise dates of symptom onset or differences in viral shedding in this population. Symptoms were assessed by nursing staff and clinicians. Of those who reported symptoms at the time of testing, 62% had cognitive impairment, while 56% of those who did not report symptoms also had cognitive impairment [44] questioning the validity of symptom reporting. Whether these findings can be generalized to other settings is unclear. Nevertheless, a focused protection approach in such special settings should be recommended.

The incremental impact on 7-day incidence may seem relatively small at 5/100,000 for 1,000,000 tests per week. However, since rapid antigen testing in the Federal Republic of Germany were in its infancy at the time of writing, we assume that 1,000,000 tests per week represent a lower limit that has already been clearly exceeded because politicians are already talking about “citizen testing” and about it being a “citizen's duty” to be tested. Furthermore, rapid antigen testing is being massively promoted by state authorities such as the Robert Koch Institute, which is subordinate to the Ministry of Health and massive public information campaigns are being announced [45]. It is therefore to be expected that the number of rapid antigen tests will increase significantly. A doubling to 2,000,000 tests would then cause an increase in the 7-day incidence by 10/100,000. The maximum calculation based on the antigen rapid test quotas given by the BGM (FMH) has made the possible massive impact on the 7-day incidence clear. In addition, there would be the SARS-CoV-2 PCR tests performed week after week, which produce false positive test results due to their error rate (specificity 98.6%). Even if the disease were no longer present (prevalence 0%) and 705,520 tests were performed from calendar week 25, 9877 false positive results would be obtained, producing an incidence of 11.9/100,000. The 7-day incidence at the end of June 2021 was around 5/100,000, lower than the predicted false-positive rates. However, the above figures include multiple testing and other methodological aspects (preselection by positive SARS-CoV-2 rapid Point-of-Care tests, high variances between laboratories due to lack of standardization of the SARS-CoV-2 PCR test; simplification of the model using averages instead of full distributions; testing in low prevalence settings). For a full explanation see Supplementary Material S2. It should also be kept in mind that testing in non-laboratory settings and by untrained people for this purpose represents an additional source of error, the quantification of which is not reliable on the basis of the data available so far. These examples clearly show how carefully the advantages and disadvantages of a population-based testing strategy must be evaluated.

Kohn et al. list several biases that can occur in diagnostic accuracy studies. In the present case, the imperfect gold standard bias and the spectrum bias are of importance. As will be shown below, the SARS-CoV-2 PCR test method is a seriously imperfect gold standard as this test is neither sufficiently standardized nor validated. As a consequence, studies repeatedly complain about low sensitivities of the rapid antigen tests even though these stem from the fact that the SARS-CoV-2 PCR test subjects with high Ct levels were classified as being test positive who have a very low, clinically irrelevant viral load, which the rapid antigen test correctly fails to detect. Therefore, some studies are moving to-wards defining Ct cut-off values for the infectivity of individuals, e.g. Ct < 30 in order to increase the sensitivity of the rapid antigen test. At the same time this concedes the clinical irrelevance of higher SARS-CoV-2 PCR Ct values so that these people would no longer be defined as infectious.

A spectrum bias results from the fact that in some clinical studies, symptomatic individuals (D+) were included in the studies (“sickest of the sick”), while individuals without symptoms (D–) were recruited (“wellest of the well”), so that sensitivity and specificity were overestimated [46] (p 1202).

In addition, the small or even non-existent role of asymptomatic and presymptomatic SARS-CoV-2 PCR test positives in the infection process, in which a high Ct value was present should be mentioned. This has now been shown in several studies [24],[34],[47],[48]. The claim in the study by Paul et al. that the viral load of asymptomatic individuals did not differ from those with symptoms is therefore surprising [6]. The construct of “asymptomatic patients” is therefore generally to be doubted from a technical point of view, also on the basis of the study from Wuhan mentioned above [24]. In this study with almost 10 million participants, there were 300 asymptomatic, SARS-CoV-2-PCR-positive patients. The examination of their 1174 closest contacts, however, yielded zero PCR-positive test results. It follows that those who test positive but are clinically asymptomatic should be considered non-infectious. This is further supported by the fact that not a single replicable SARS-CoV-2 virus could be grown from the 300 positive samples of asymptomatic virus cultures in the study.

In its new Recommendations for national SARS-CoV-2 testing strategies and diagnostic capacities, the WHO recommends discontinuing mass testing of asymptomatic persons due to the lack of evidence and the costs involved. This is only recommended for frequently exposed groups such as health care workers or long-term care facility workers. It is stated that the evidence base on effectiveness of widespread asymptomatic population screening and self-testing for SARS-CoV-2 is currently lacking and therefore is not sufficient to make recommendations [23].

### 4.2. Imperfect gold standard bias

Imperfect gold standard bias is an epidemiological term discussed by Kohn, Carpenter & Newman [46]. This technical term implies that the criterion against which other tests are validated has itself questionable quality criteria, so that the unfavorable quality criteria of the tests under investigation, which may be derived from this, are improperly criticized.

Since the use of the SARS-CoV-2 PCR test method has been criticized as the gold standard for validating the rapid antigen tests, some empirical results regarding the SARS-CoV-2 PCR test method will be discussed.

The importance of Ct values in the SARS-CoV-2 PCR test method has already been pointed out. With Ct values between 10 and 20 viruses were also detectable in the cell culture in 76.6% of the samples. The proportion of culture-positive cell samples decreased to 24.1% for Ct values between 20 and 30 and to 2.9% for Ct values >30 [49]. Individuals with higher Ct values, in whom viruses could be cultured, had corresponding clinical symptoms. In their review, Jefferson et al. conclude that virus cultivation in cell cultures was rarely possible at Ct values >24 or when the onset of symptoms was more than 8 days ago [50]. This was confirmed in another study [39]. The contagiousness of people with Ct values >24 and duration of symptoms >8 days is therefore very likely to be low, if still present at all. Rao et al. come to fundamentally similar conclusions in their review and advocate that Ct values should always be given in PCR testing, as these can be used to make important clinical and health policy decisions [51].

The expressed criticism of the pseudo-validation of the rapid antigen tests by the SARS-CoV-2 PCR test method is supported in a commentary in the renowned journal Lancet [7]. The authors argue that the SARS-CoV-2 PCR test method is an inappropriate gold standard to validate the SARS-CoV-2 rapid antigen test. The SARS-CoV-2 PCR test detects RNA even from past infections in people who are no longer symptomatic. The median time of positive results in the PCR test is 22–33 days, while most infected persons are infectious for 4–8 days. Consequently, 50–75% of PCR test positives are post-infectious and thus not acutely infectious. As a result, they should not be included in the official statistics at all. PCR tests can detect virus concentrations that are irrelevant for an infection and still show positive results weeks to months after an infection. Therefore, the authors are not surprised that the rapid antigen tests have low sensitivities compared to the SARS-CoV-2 PCR tests as they do not detect those with low viral loads. For a test from which health policy decisions are derived, this means that it has fulfilled its task absolutely correctly [7] (p 1426). It should merely be added that, due to the equally imperfect specificities of the rapid antigen tests, narrow indications must be made, and they should never be used as mass tests as this in turn leads to false positive results (i.e. with a self-reinforcing effect, see model calculations in the results section).

Surkova et al. note that no gold standard exists for validating the SARS-CoV-2 PCR test [52]. Rates of false positive test results in the UK ranged from 0.8% to 4.0% in autumn 2020. If there is a low pretest probability, test results should be interpreted with caution and a second sample should be taken. Lühmann reports low positive predictive values for the SARS-CoV-2 PCR test in mass testing with low prevalence [53]. The probability of actually having the disease in the case of a positive test result is therefore low. In the diagnosis of SARS-CoV-2, the WHO points out that testing for other pathogens should also be carried out. Co-infections with other pathogens do not exclude COVID-19 and vice versa. In particular, co-infections with influenza A have been reported. Therefore, a well-founded differential diagnosis is to be recommended [54]. In an information notice of January 2021, the WHO clearly stated that the Ct is inversely proportional to the viral load, as also shown in the above studies and reviews. A new sample should be taken if the test results do not match the clinical picture and consequently a new PCR test should be performed. Furthermore, the WHO states that the prevalence of a disease influences the predictive diagnostic value. The lower the prevalence the higher the risk of false positive results. This means that the likelihood that a person who receives a positive PCR test result is actually infected with SARS-CoV-2 decreases as prevalence decreases regardless of the specificity adopted [36]. The WHO goes on to instruct clearly that PCR tests should be considered diagnostic aids only. Consequently, medical personnel must assess each result in the context of the timing of sample collection, type of sample, clinical observations, patient history, confirmed contacts and epidemiological information. The sole assessment of a positive SARS-CoV-2 PCR test as infectious is therefore inadmissible, not least on the basis of the Infection Protection Act. A decision as to whether an infection is actually present can and may only be made by a physician.

This is further supported by a recent German study. In an evaluation of SARS-CoV-2 PCR test results from a laboratory serving about 80% of the German city of Münster with a population of about 313,000, only 40.6% of the positive subjects had Ct values <25, which were considered infectious in the UK Office for National Statistics (ONS) COVID-19 household survey [40]. Therefore, it is not suitable to simply count positive SARS-CoV-2 PCR test results to represent the infectiousness of the disease which are then used to operationalize a disease occurrence in the context of an incidence parameter. It should further be mentioned that there is no standardized testing strategy in Germany. This means that already the daily incidence values are not comparable. Furthermore, the total number of tests carried out per week in Germany cannot be reliably determined, as only a certain number of laboratories report them voluntarily to the RKI.

Another difficulty in validating both rapid antigen test results and SARS-CoV-2 PCR test results are interpretation problems with the corresponding virus cultures. There is no direct evidence of the relationship between infectivity in cell cultures and virus transmissibility in humans. It is unknown whether individuals with low viral loads who test positive in virus cultures contribute to virus spread. Thus, it may be that cell culture-based testing is too sensitive a method to adequately represent viral transmission in humans. Furthermore, the detection rate of rapid antigen tests is thought to be affected in the context of natural mutations [55]. Samples with Ct values >33 can only rarely cause cultivation in virus cultures, which means low infectivity [56]. In another study, no virus cultivation was achieved in samples with Ct values >29 [37]. On the other hand, other authors state that Ct values between 17 and 32 represent culturable virus amounts and should be assumed to be infectious but mention in the same article that it is not yet known how many SARS-CoV-2 virions are required to cause an infection [41]. Thus, this method represents a suitable gold standard for the presence of replicable viruses, which, however, does not necessarily mean a gold standard for infectivity at the same time.

### 4.3. Methodological problems of repeated screening tests

Paul et al. cite recommendations that repeated testing with rapid antigen tests every 2–3 days is appropriate [6], but this is based on modelling that has often been shown to be inaccurate for COVID-19 [57]. The authors conclude that the value of rapid antigen testing as a single test in asymptomatic individuals is significantly limited [6] (p 14).

For example, modelling exists to show that the effectiveness of screening is largely based on the frequency of testing. The authors admit that this leads to some false positive results, which result in unnecessary quarantine [58], but this is not discussed further. The modelling is not based on real samples but on constructed samples and only the sensitivity of rapid tests is considered, not the specificity.

All these calls for regular, repeated screening do not take into account crucial methodological considerations. Indeed, it is obvious that the probability of a false positive result increases even further with an increasing number of screening tests [59],[60]. Other mathematical models would therefore be necessary for an adequate interpretation of the results of repeated measurements with rapid antigen tests on the same persons. Dinnes et al. consequently criticize that in repeated testing the entire test strategy would have to be examined and not only the tests used [4].

Iacobucci criticizes the repeated mass testing at British universities, as it only produced a low positive rate of 0.5%. Political goals had taken precedence over those of science or health [61]. Many false positive results were produced at enormous cost. Massive doubts are expressed about the scientificity and ethical justification of the testing programme and recommendations are made to stop it. Instead, only students with corresponding symptoms should be tested. Incidentally, this is the only possible logical consequence of the above-mentioned finding from Wuhan that asymptomatic individuals are unlikely to cause an infection and this also corresponds to the recent recommendations of the WHO [23].

## 5. Conclusions

Both rapid antigen tests and the SARS-CoV-2 PCR test method should only be used if there is a corresponding pre-test probability, i.e. with a corresponding diagnostic hypothesis. In effect, this means that they should be used on people suffering from respiratory symptoms and not on symptom-free people [23] because, even if the disease disappears completely, the resulting test pandemic will never end due to false positive results.

In general, neither the pseudo-validation of the rapid antigen tests on a procedure such as the SARS-CoV-2 PCR test method nor the test rationale behind it is convincing from a test-theoretical and evidence-based perspective:

The preselection of positive test results by the rapid antigen tests leads to an increased pre-test probability during post-testing by the SARS-CoV-2 PCR tests. This in turn increases the positive rate of the SARS-CoV-2 PCR tests, which is only an effect of the increased testing and corresponding pre-selection by the rapid antigen tests. This, however, is then interpreted by the political mandate holders as a worsening of the infectious situation irrespective of the increased number of tests. The positive rate is used by politicians as an indicator of the severity of the epidemic situation. If the number of tests exerts an influence on the assessment of this situation, this is an inadmissible conclusion. Furthermore, this parameter is inappropriate because it is based on a non-representative population sample each week. A further complication is the fact that the test strategy and the number of tests per 100,000 varies regionally in a significant manner so that comparisons between regions are only of limited value and the average positive rate for Germany may be distorted by the absence of standardization.

Furthermore, a differential diagnosis would be required e.g., by means of a multiplex PCR test (e.g. SARS-CoV-2-qPCR Plus). This would detect the new coronavirus with influenza A and B as well as RSV A and B and other particularly frequent respiratory tract pathogens, instead of just screening one pathogen. It can be concluded that the practical benefit of mass testing with rapid antigen tests cannot be proven [23].

Current data on the global Infection Fatality Rate (IFR) for COVID-19 is 0.15% [62], which means that 99.85% of those with a COVID-19 infection survive the disease. In view of such a comparatively low mortality rate and correspondingly high survival rate, the mass testing described in this context is not expedient from an epidemiological aspect and is not justified.

## References

[1] Protzer U, Keppler O, Renz H (2021). Positionspapier des Netzwerk B-FAST im Nationalen Forschungsnetzwerk der Universitätsmedizin zu COVID-19 zur Anwendung und Zulassungspraxis von Antigen-Schnelltests zum Nachweis des neuen Coronavirus, SARS-CoV-2.

[2] European Centre for Disease Prevention and Control (2020). Options for the use of rapid antigen tests for COVID-19 in the EU/EEA and the UK. Tech Rep.

[3] Robert Koch Institut (RKI) Corona-Schnelltest-Ergebnisse verstehen, Berlin, 2021.

[4] Dinnes J, Deeks JJ, Berhane S (2021). Rapid, point-of-care antigen and molecular-based tests for diagnosis of SARS-CoV-2 infection. Cochrane Database Syst Rev.

[5] Riccò M, Ferraro P, Gualerzi G (2020). Point-of-care diagnostic tests for detecting SARS-CoV-2 antibodies: a systematic review and meta-analysis of real-world data. J Clin Med.

[6] Robert Koch Institut (RKI) Epidemiologisches Bulletin, 2021.

[7] Mina MJ, Peto TE, García-Fiñana M (2021). Clarifying the evidence on SARS-CoV-2 antigen rapid tests in public health responses to COVID-19. Lancet.

[8] Corman VM, Landt O, Kaiser M (2020). Detection of 2019 novel coronavirus (2019-nCoV) by real-time RT-PCR. Euro Surveill.

[9] Zeichhardt H, Kammel M (2020). Kommentar zum Extra Ringversuch Gruppe 340 Virusgenom-Nachweis-SARS-CoV-2. Düsseldorf.

[10] Scohy A, Anantharajah A, Bodéus M (2020). Low performance of rapid antigen detection test as frontline testing for COVID-19 diagnosis. J Clin Virol.

[11] Pray IW, Ford L, Cole D (2021). Performance of an antigen-based test for asymptomatic and symptomatic SARS-CoV-2 testing at two University campuses - Wisconsin, September-October 2020. MMWR Morb Mortal Wkly Rep.

[12] Robert Koch Institut (RKI) Antworten auf häufig gestellte Fragen zum Coronavirus SARS-CoV-2/Krankheit COVID-19, 2021. Werden die Meldedaten durch die wachsende Anzahl an Schnelltests verzerrt?.

[13] Salzberger B, Buder F, Lampl BT (2020). Epidemiologie von SARS-CoV-2/COVID-19. Gastroenterologe.

[14] Schüller K Statistikerin: Positive Schnelltests sind meist falsch – selbst wenn sie Mediziner durchführen, 2021.

[15] Pouwels KB, House T, Pritchard E (2021). Community prevalence of SARS-CoV-2 in England from April to November, 2020: results from the ONS Coronavirus Infection Survey. Lancet Public Health.

[16] Vodičar PM, Oštrbenk Valenčak A, Zupan B (2020). Low prevalence of active COVID-19 in Slovenia: a nationwide population study of a probability-based sample. Clin Microbiol Infect.

[17] Gudbjartsson DF, Helgason A, Jonsson H (2020). Spread of SARS-CoV-2 in the Icelandic population. N Engl J Med.

[18] Snoeck CJ, Vaillant M, Abdelrahman T (2020). Prevalence of SARS-CoV-2 infection in the Luxembourgish population: the CON-VINCE study. medRxiv.

[19] Robert Koch Institut (RKI) Täglicher Lagebericht des RKI zur Coronavirus-Krankheit-2019 (COVID-19), Berlin, 2021.

[20] Bundesministerium für Gesundheit (German Ministry of Health) Fragen und Antworten zu Schnell- und Selbsttests zum Nachweis von SARS-CoV-2, 2021.

[21] Statistisches Bundesamt Bevölkerungsstand, 2021.

[22] Grobbee DE, Hoes AW (2014). Clinical epidemiology: principles, methods, and applications for clinical research.

[23] World Health Organisation Recommendations for national SARS-CoV-2 testing strategies and diagnostic capacities. Interim guidance, Geneva, 2021.

[24] Cao S, Gan Y, Wang C (2020). Post-lockdown SARS-CoV-2 nucleic acid screening in nearly ten million residents of Wuhan, China. Nat Commun.

[25] Day M (2020). Covid-19: four fifths of cases are asymptomatic, China figures indicate. BMJ.

[26] Obi OC, Odoh DA (2021). Transmission of coronavirus (SARS-CoV-2) by presymptomatic and asymptomatic COVID-19 carriers: a systematic review. Eur J Med Educ Technol.

[27] Maniaci A, Iannella G, Vicini C (2020). A case of COVID-19 with late-onset rash and transient loss of taste and smell in a 15-year-old boy. Am J Case Rep.

[28] Li DTS, Samaranayake LP, Leung YY (2021). Facial protection in the era of COVID-19: a narrative review. Oral Dis.

[29] Rothe C, Schunk M, Sothmann P (2020). Transmission of 2019-nCoV Infection from an asymptomatic contact in Germany. N Engl J Med.

[30] Day M (2020). Covid-19: identifying and isolating asymptomatic people helped eliminate virus in Italian village. BMJ.

[31] Lubrano R, Bloise S, Testa A (2021). Assessment of respiratory function in infants and young children wearing face masks during the COVID-19 pandemic. JAMA Netw Open.

[32] Jefferson T, Spencer EA, Brassey J (2021). Transmission of severe acute respiratory syndrome coronavirus-2 (SARS-CoV-2) from pre and asymptomatic infected individuals. A systematic review. Clin Microbiol Infect.

[33] Savvides C, Siegel R (2020). Asymptomatic and presymptomatic transmission of SARS-CoV-2: a systematic review. medRxiv.

[34] Qiu X, Nergiz AI, Maraolo AE (2021). Defining the role of asymptomatic and pre-symptomatic SARS-CoV-2 transmission-a living systematic review. Clin Microbiol Infect.

[35] Seifried J, Böttcher S, Oh DY (2021). Was ist bei Antigentests zur Eigenanwendung (Selbsttests) zum Nachweis von SARS-CoV-2 zu beachten?. Epidemiologisches Bull.

[36] World Health Organisation WHO Information Notice for IVD Users 2020/05. Nucleic acid testing (NAT) technologies that use polymerase chain reaction (PCR) for detection of SARS-CoV-2, 2021.

[37] Strömer A, Rose R, Schäfer M (2020). Performance of a point-of-care test for the rapid detection of SARS-CoV-2 antigen. Microorganisms.

[38] Iglὁi Z, Velzing J, van Beek J (2021). Clinical evaluation of Roche SD Biosensor rapid antigen test for SARS-CoV-2 in Municipal Health Service Testing Site, the Netherlands. Emerg Infect Dis.

[39] Bullard J, Dust K, Funk D (2020). Predicting infectious severe acute respiratory syndrome coronavirus 2 from diagnostic samples. Clin Infect Dis.

[40] Stang A, Robers J, Schonert B (2021). The performance of the SARS-CoV-2 RT-PCR test as a tool for detecting SARS-CoV-2 infection in the population. J Infect.

[41] Platten M, Hoffmann D, Grosser R (2021). SARS-CoV-2, CT-values, and infectivity-conclusions to be drawn from side observations. Viruses.

[42] RWI – Leibniz-Institut für Wirtschaftsforschung Analysen zum Leistungsgeschehen der Krankenhäuser und zur Ausgleichspauschale in der Corona-Krise. Ergebnisse für den Zeitraum Januar bis Dezember 2020 Im Auftrag des Bundesministeriums für Gesundheit, Essen, 2021.

[43] Kowall B, Standl F, Oesterling F (2021). Excess mortality due to Covid-19? A comparison of total mortality in 2020 with total mortality in 2016 to 2019 in Germany, Sweden and Spain. PloS One.

[44] Arons MM, Hatfield KM, Reddy SC (2020). Presymptomatic SARS-CoV-2 infections and transmission in a skilled nursing facility. N Engl J Med.

[45] Seifried J, Böttcher S, von Kleist M (2021). Antigentests als ergänzendes instrument in der Pandemiebekämpfung. Epidemiologisches Bull.

[46] Kohn MA, Carpenter CR, Newman TB (2013). Understanding the direction of bias in studies of diagnostic test accuracy. Acad Emerg Med.

[47] Cheng HY, Jian SW, Liu DP (2020). Contact tracing assessment of COVID-19 transmission dynamics in Taiwan and risk at different exposure periods before and after symptom onset. JAMA Intern Med.

[48] Madewell ZJ, Yang Y, Longini IM (2020). Household transmission of SARS-CoV-2: a systematic review and meta-analysis. JAMA Netw Open.

[49] Gniazdowski V, Morris CP, Wohl S (2021). Repeat coronavirus disease 2019 molecular testing: correlation of SARS-CoV-2 culture with molecular assays and cycle thresholds. Clin Infect Dis.

[50] Jefferson T, Spencer EA, Brassey J (2020). Viral cultures for COVID-19 infectious potential assessment - a systematic review. Clin Infect Dis.

[51] Rao SN, Manissero D, Steele VR (2020). A systematic review of the clinical utility of cycle threshold values in the context of COVID-19. Infect Dis Ther.

[52] Surkova E, Nikolayevskyy V, Drobniewski F (2020). False-positive COVID-19 results: hidden problems and costs. Lancet Respir Med.

[53] Lühmann D (2020). Anlassloses Testen auf SARS-Cov-2. Für Personen, bei denen kein begründeter Verdacht auf eine Infektion vorliegt, ist die Aussagekraft eines einzelnen positiven Testergebnisses verschwindend gering. KVH J.

[54] World Health Organisation Diagnostic testing for SARS-CoV-2. Interim guidance, Geneva, 2020.

[55] Kohmer N, Toptan T, Pallas C (2021). The comparative clinical performance of four SARS-CoV-2 rapid antigen tests and their correlation to infectivity in vitro. J Clin Med.

[56] Mboumba Bouassa RS, Veyer D, Péré H (2021). Analytical performances of the point-of-care SIENNA™ COVID-19 Antigen Rapid Test for the detection of SARS-CoV-2 nucleocapsid protein in nasopharyngeal swabs: a prospective evaluation during the COVID-19 second wave in France. Int J Infect Dis.

[57] Ioannidis JPA, Cripps S, Tanner MA (2020). Forecasting for COVID-19 has failed. Int J Forecast.

[58] Larremore DB, Wilder B, Lester E (2021). Test sensitivity is secondary to frequency and turnaround time for COVID-19 screening. Sci Adv.

[59] Gelfand AE, Wang F (2000). Modelling the cumulative risk for a false-positive under repeated screening events. Stat Med.

[60] Thompson ML (2003). Assessing the diagnostic accuracy of a sequence of tests. Biostatistics.

[61] Iacobucci G (2021). Covid-19: mass testing at UK universities is haphazard and unscientific, finds BMJ investigation. BMJ.

[62] Ioannidis JPA (2021). Reconciling estimates of global spread and infection fatality rates of COVID-19: an overview of systematic evaluations. Eur J Clin Invest.

