# Supplementary material for: Methodological problems of SARS-CoV-2 rapid point-of-care tests when used in mass testing
Source: AIMS Public Health. 2021 Nov 23;9(1):73–93. doi: 10.3934/publichealth.2022007 (PMC8755956; doi:10.3934/publichealth.2022007)
Supplement: Supplementary file 1 [file publichealth-09-01-007-s001.pdf]

Public Health

http://www.aimspress.com/journal/aimsph

AIMS Public Health, 9(1): 73–93.
DOI: 10.3934/publichealth.2022007
Received: 17 September 2021
Accepted: 16 November 2021
Published: 23 November 2021

Research article

Methodological problems of SARS-CoV-2 rapid point-of-care tests when used in mass testing

Oliver Hirsch[1,*], Werner Bergholz[2], Kai Kisielinski[3], Paul Giboni[4] and Andreas Sönnichsen[5]

[1] Department of Psychology, FOM University of Applied Sciences, Birlenbacher Str. 17, 57078 Siegen, Germany
[2] International Standards Consulting GmbH, 30989 Gehrden, Germany
[3] Private Practice, 40212 Düsseldorf, Germany
[4] Private Practice, 22763 Hamburg, Germany
[5] Department of General Practice and Family Medicine, Center for Public Health, Medical University of Vienna, Wien, Austria

* **Correspondence:** Email: oliver.hirsch@fom.de.

Supplementary Material S1

Detailed discussion of studies on rapid point-of-care tests

In the instructions for the SARS-CoV-2 Rapid Antigen Test of the company SD Biosensor from South Korea, distributed by Roche, studies performed in Brazil and India report a sensitivity of 96.52% and a specificity of 99.68%. However, positive test results would not exclude co-infections with other pathogens. Consequently, if co-infection with influenza A were to be present, the contribution of the single pathogen to any symptomatology could not be inferred. Positive test results would not differentiate between SARS-CoV-2 and SARS-CoV-1.

The SARS-CoV-2 Rapid Antigen Test from Hotgen is reported to have a sensitivity of 95.37% and a specificity of 99.13% on n = 223 samples, without giving a citation for the investigation in the package insert. However, there was a very high prevalence of 48.4% positive SARS-CoV-2 PCR test results in this sample.

The SD Biosensor test was compared using a supervised self-collected nasal mid-turbinate (NMT) versus a professional-collected nasopharyngeal (NP) swab [1]. Of the 289 participants at a

university hospital testing centre, only 39 (13.5%) tested positive for SARS-CoV-2 by RT-PCR although 97.6% showed symptoms that would have been consistent with COVID-19. NMT sampling showed a sensitivity of 74.4% (95% CI 58.9–85.4%) and a specificity of 99.2% (95% CI 97.1–99.8%) compared with RT-PCR. The sensitivity of NP sampling was 79.5% (95% CI 64.5–89.2%) and the specificity was 99.6% (95% CI 97.8–100%).

The Panbio™ coronavirus disease 2019 (COVID-19) Ag Rapid Test Device (PanbioRT) (Abbott Diagnostic GmbH, Jena, Germany) was evaluated for its performance criteria in a pre-selected sample of suspected COVID-19 cases in ten Spanish university hospitals [2]. Samples from a total of n = 958 patients were collected and analyzed by trained professionals. Of these, 359 (37.5%) were positive in RT-PCR. The sensitivity was 90.5% and the specificity was 98.8%.

Three lateral flow assays (RIDA®QUICK SARS-CoV-2 Antigen (R-Biopharm), SARS-CoV-2 Rapid Antigen Test (Roche)), and NADAL® COVID-19 Ag Test (Nal von Minden GmbH, Regensburg, Germany) and a microfluidic immunofluorescence assay (SARS-CoV-2 Ag Test (LumiraDx GmbH, Cologne, Germany)) were tested on 100 clinical samples, 74 of which tested positive in RT-PCR using ORF1 as the target gene. Sensitivity varied between 24.3% and 50%. Specificity was reported between 96.2% and 100%. Only 51.6% of the SARS-CoV-2 PCR test positive samples were infectious in the cell cultures [3].

In two university hospitals in Munich, the SARS-CoV-2 Rapid Antigen Test of SD Biosensor from South Korea, distributed by Roche, and the SD Biosensor Standard F COVID-19 Ag FIA (FIA) were tested [4]. The test was carried out by laboratory personnel. A sensitivity of 50.3% and a specificity of 97.7% was determined for the former and a sensitivity of 45.4% and a specificity of 97.8% for the FIA. The median Ct value of the SARS-CoV-2 PCR test for the positive rapid antigen tests was 23.8 and that of the negative rapid antigen tests was 33.8–34.0, which again underlines the importance of standardizing the SARS-CoV-2 PCR test and establishing a cut-off value for the clinical relevance of the Ct value. The sensitivities of the rapid antigen tests examined were again significantly lower than the values stated by the manufacturers. Consequently, the tests detect individuals with a high viral load. The positive predictive value (PPV), i.e. the probability of actually being SARS-CoV-2 positive with a positive test result, was only about 2%. False positives lead to unnecessary quarantine measures and cause emotional distress. The authors express concern that in their sample about 40% of SARS-CoV-2 PCR test positives patients would have received a negative result, whereby the lack of a gold standard for the SARS-CoV-2 PCR test and the high Ct values must also be taken into account. The authors argue against mass use by laypersons as in their opinion the disadvantages would outweigh the advantages [4].

Cerutti et al. collected the quality criteria on patients in an emergency room and on travelers returning home [5]. Unfortunately, some terms are not correctly interpreted or presented by the authors. Moreover, the values presented are surprising, in part unrealistically high compared to the other studies reviewed: sensitivity 87.4%, specificity 100%, positive predictive value 70.6%, negative predictive value 100%. Figure 1 of the publication shows that with the rapid antigen test, some people with Ct values >25 in the PCR test were tested positive. According to further studies, these have such a low viral load that they are to be classified as non-infectious. This shows the pseudo-validation in the existing studies in that both test methods identify people as false positive. This results in corresponding negative individual, social and health policy implications, yet this is considered a good match.

Corman et al. compared 7 different rapid antigen tests [6]. This study is not really comparable with the currently cited ones as it was a case-control design and used stored samples from symptomatic patients resulting in specificities of 88.24% to 100%. Self-testing was performed in 35 healthy individuals with specificities ranging from 82.86% to 100%. It is inferred that rapid antigen tests are capable of reliably detecting COVID-19 disease within the first week. Screening asymptomatic individuals to show the absence of the virus is more difficult. It should be noted that patients with a high viral load will have corresponding symptoms and appropriate differential diagnostics should be carried out for them.

The investigation of three antigen rapid tests performed in the outpatient setting by professionals (Bioeasy 2019-nCoV Ag Fluorescence Rapid Test Kit, COVID-19 Ag Respi-Strip by Coris Bioconcept, STANDARD Q COVID-19 Ag Test by SD Biosensor) yielded different quality criteria [7]. A total of n = 2417 individuals were involved in the study, including at its drive-in test station and an outpatient test facility, of whom n = 70 (2.9%) had a positive SARS-CoV-2 PCR test result. It further states that of these only n = 30 (42.9%) had Ct values <25 and consequently a suitably high viral load to be considered infectious. This in turn has implications for a test to be validated on such a trial [8], as it raises the question of the clinical relevance of test results. The SD Biosensor rapid antigen test had a sensitivity of 76.6% and a specificity of 99.3%, the Bioeasy test had a sensitivity of 66.7% and a specificity of 93.1%, and the Coris test had a sensitivity of 50% and a specificity of 95.8%. The authors report that false positives were followed up and they concluded that there were unknown cross-reactions. This again highlights the importance of differential diagnosis. The authors conclude that the rapid antigen tests can identify people with high viral loads, but that more research is needed on their implementation.

The study by Scohy et al. [9] highlights the problem of validating the rapid antigen tests on the SARS-CoV-2 PCR test . A low sensitivity of 30.2% is criticized. The sensitivity of the rapid antigen test would drop significantly if low viral loads were present at Ct values above 30. This is understandable, since people with such high Ct values are not to be regarded as infectious. The methodological error here is that the SARS-CoV-2 PCR test is used as the gold standard and thus it is assumed that results with such a high Ct value are to be classified as positive cases, which the rapid antigen test then has to find. This is to be regarded as theoretically and substantially erroneous.

In a public place in San Francisco, USA, a total of n = 878 individuals were tested by a laboratory worker in each case with the BinaxNOWTM Abbott COVID-19 Ag card and with a RT-PCR test [10]. Within this sample, 3% tested positive with the RT-PCR test. The authors describe that they evaluated the tests on the first day of the study according to the manufacturer's criteria. According to the published data this showed a sensitivity of 71.4%, a specificity of 95.7%, a positive predictive value of 35.7% and a negative predictive value of 99%. After changing the reading criteria for a positive result, together with the RT-PCR test definition of a Ct value <30 for a positive result, a sensitivity of 93.3% and a specificity of 99.9% were then reported.

The Lumipulse® Antigen Test was investigated in two samples with regard to its quality criteria [11]. The first sample consisted of n = 226 selected persons with suspected SARS-CoV-2 infection, the second sample of n = 1778 unselected persons from schools, prisons, nursing homes and hospitals. Individuals with Ct values >35 in the SARS-CoV-2 PCR test were considered test negative. In the selective sample, the prevalence was 42%, the sensitivity was 92.6%, and the specificity was 90.8%. In the screening sample, the prevalence was 5.2%, the sensitivity was 100%, the specificity 94.8%. Despite the problematic specificities, the authors

advocate the use of the test in mass testing and justify this with the high sensitivities and the high negative predictive values [11] (p 395).

Other international studies that examined samples collected and analyzed by professionals at relatively high prevalences achieved specificities in the range of 99.9–100% under these fairly ideal conditions [12–14].

Detailed calculations from the introduction

The results for asymptomatic patients (sensitivity 58.1%, specificity 98.9%) from the Cochrane review [15] are used. Applied to a sequential mass testing strategy with a prevalence of 0.5%, there are 10,945 false positive test results (Figure S1). The positive predictive value is 20.97%, which means that 20.97% of those who test positive are actually positive for SARS-CoV-2. The negative predictive value (NPV) is 99.79%, which means that 99.79% of the individuals for whom a negative test result is indicated are actually considered negative for SARS-CoV-2.

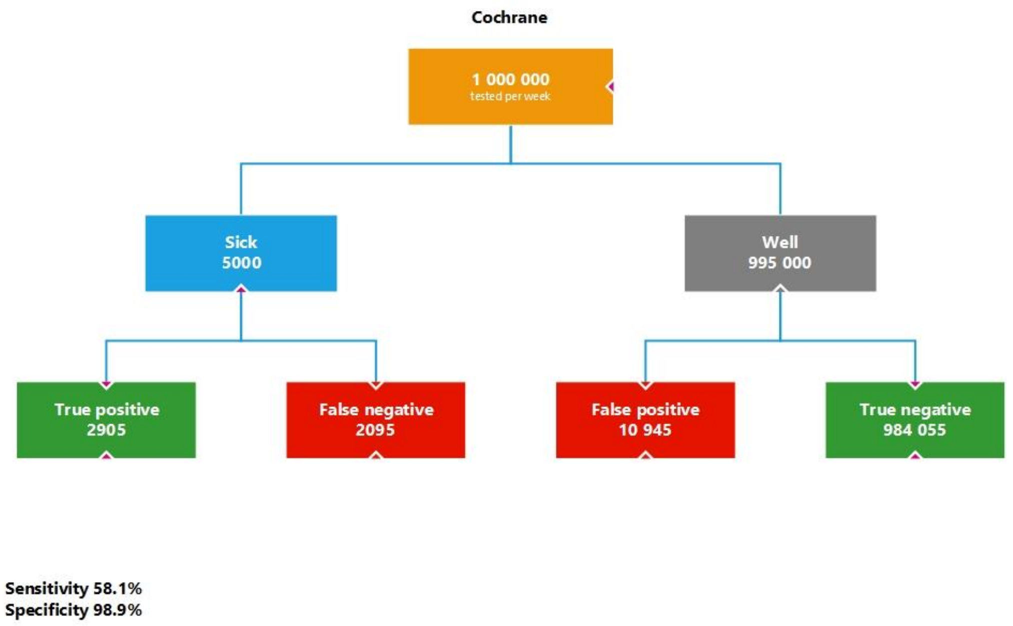

Figure S1. Simulation calculation for average quality criteria of asymptomatic patients in the Cochrane review [15] with sequential testing strategy and a prevalence of 0.5%.

If the 13,850 people identified as positive (2905 true positives + 10,945 false positives) are now retested with a SARS-CoV-2 PCR test (sensitivity 99.7%, specificity 98.6%) a prevalence of 20.97% is assumed as this is the pre-test probability to the extent of the PPV. Under these assumptions, 153 would still be false positives who would be wrongfully quarantined. The additional rapid antigen tests would increase the 7-day incidence by (2897 + 153)/831.9 = 3.67/100,000 (Figure S2).

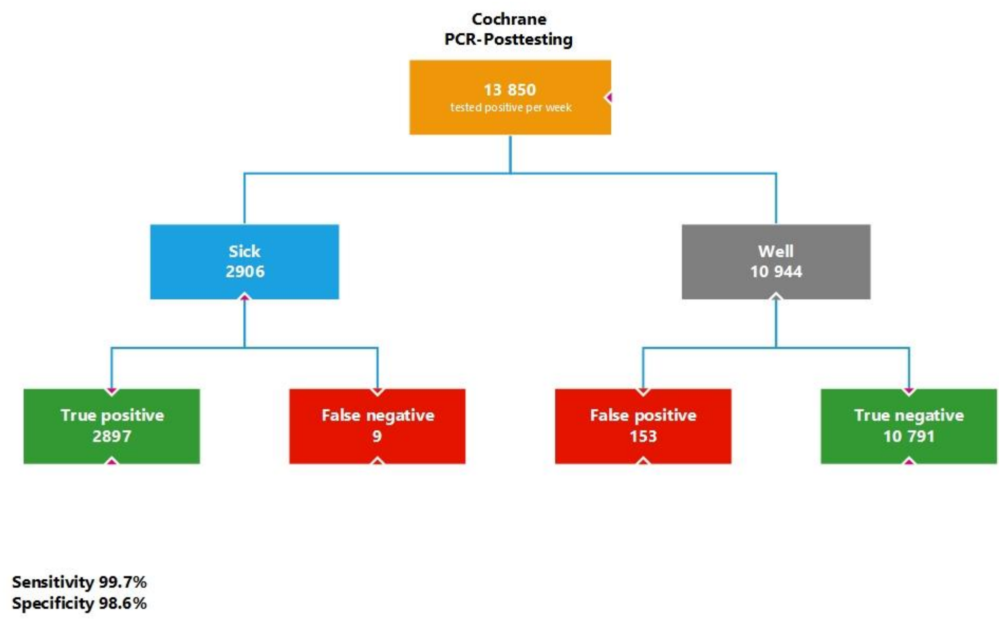

FigureS2. Simulation calculation for PCR post-testing of positive test results of asymptomatic people from the Cochrane review [15] from Figure A3 with a pre-test probability of 20.98%.

A third example are the results of mass testing at two universities in Wisconsin [16]. A sensitivity of 41.2% and a specificity of 98.4% were reported. Applied to a sequential mass testing strategy with a prevalence of 0.5%, there are 15,920 false positive test results (Figure S3). The positive predictive value is 11.46%, which means that 11.46% people who test positive are actually positive for SARS-CoV-2. The negative predictive value (NPV) is 99.70%, which means that 99.70% of those who test negative are actually considered negative for SARS-CoV-2.

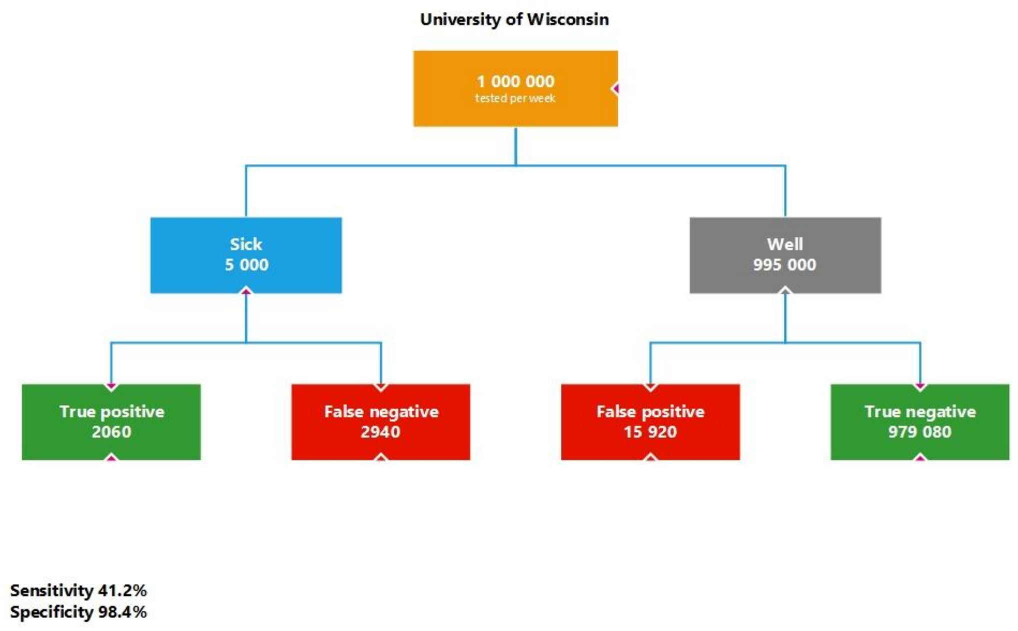

Figure S3. Simulation calculation for the University of Wisconsin data [16] with sequential testing strategy and prevalence of 0.5%.

If the 17,980 individuals identified as positive (2060 true positives + 15,920 false positives) are now retested with a SARS-CoV-2 PCR test (sensitivity 99.7%, specificity 98.6%), a prevalence of 11.46% is assumed, since this is the pre-test probability in the range of the PPV. Based on these hypotheses there would still be 223 false positives who would be unjustly sent to quarantine. The additional rapid antigen tests would increase the 7-day incidence by (2054 + 223)/831.9 = 2.74/100,000 (Figure S4).

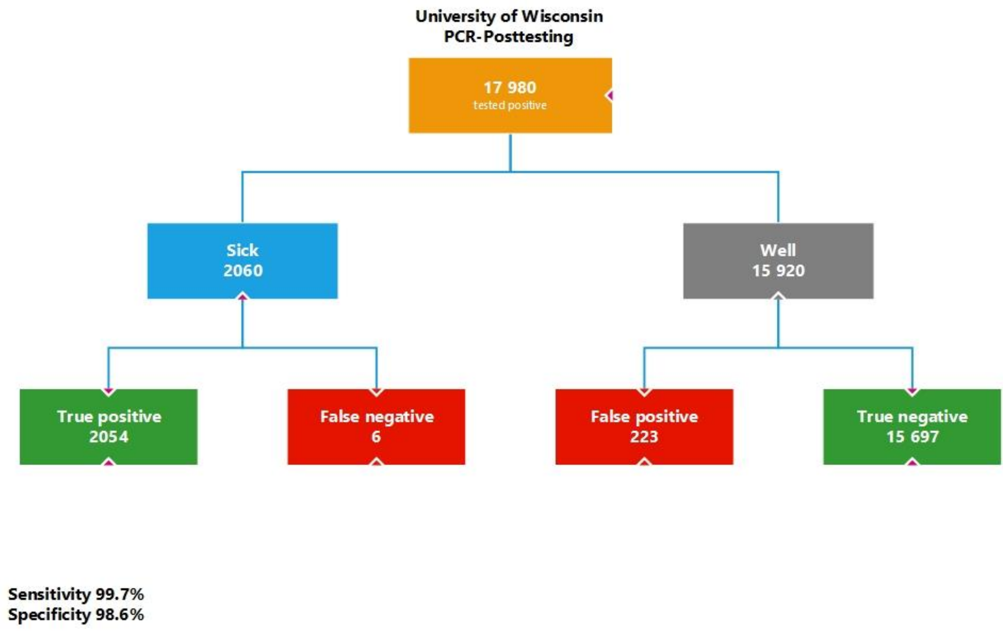

Figure S4. Simulation calculation for PCR post-testing of positive test results using the University of Wisconsin Figure S3 data with a pre-test probability of 11.46%.

The fourth example uses the values in the information document of the Robert Koch Institute (RKI) [17]. A sensitivity of 80% and a specificity of 98% are given. Applied to a sequential mass testing strategy with a prevalence of 0.5%, 19,900 false positive test results are obtained (Figure S5). The positive predictive value is 16.74%, which means that 16.74% of persons who test positive are actually positive for SARS-CoV-2. The negative predictive value (NPV) is 99.90%, which means that 99.90% of those who test negative are actually considered negative for SARS-CoV-2.

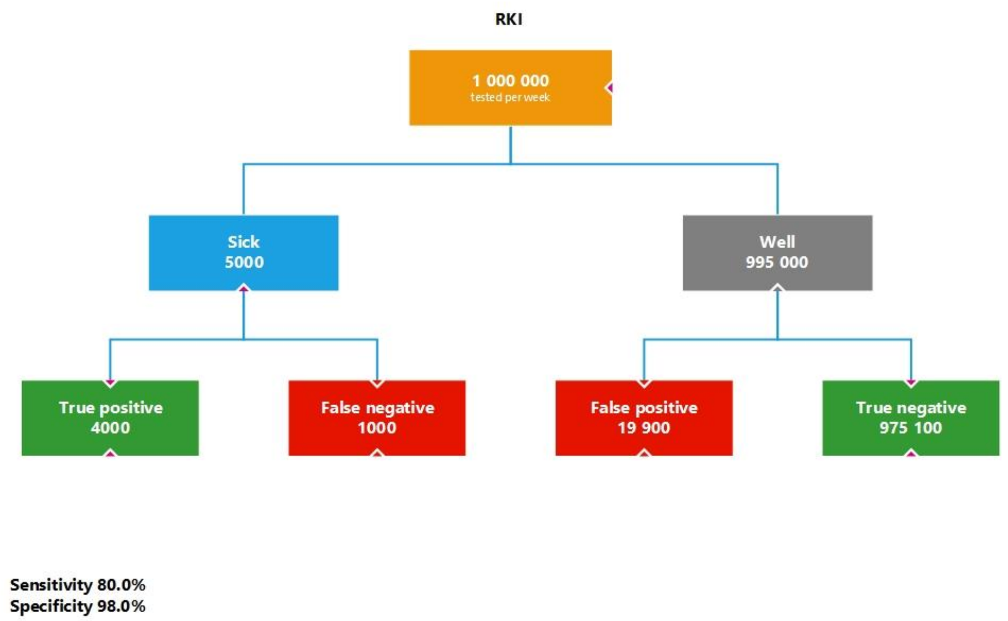

Figure S5. Simulation calculation for the data from the RKI information document [17] with a sequential testing strategy and prevalence of 0.5%.

If the 23,900 people identified as positive (4000 true positives + 19,900 false positives) are now retested with a SARS-CoV-2 PCR test (sensitivity 99.7%, specificity 98.6%), a prevalence of 16.74% is assumed, as this is the pre-test probability at the level of the PPV. Based on these assumptions there would still be 279 individuals as false positives who would be unjustifiably quarantined. The additional rapid anti-gene tests would increase the 7-day incidence by (3989 + 279)/831.9 = 5.13/100,000 (Figure S6).

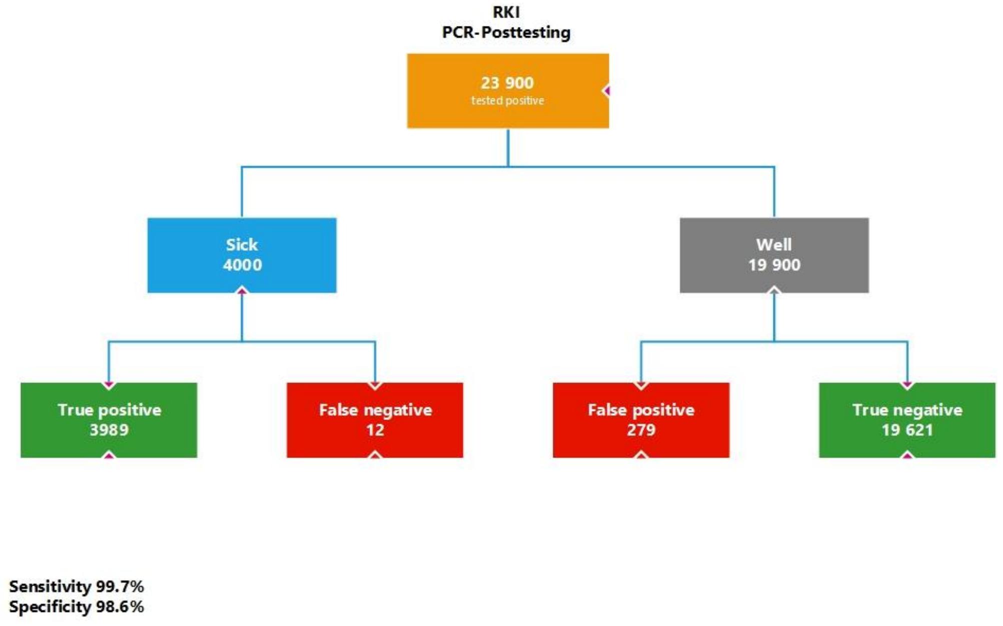

Figure S6. Simulation calculation for the data from the RKI information document from Figure S5 with a pre-test probability of 16.74%.

Finally, a previous maximum calculation is to be made using the contingent data for self-tests from the German Federal Ministry of Health [18]. A sensitivity of 80% and a specificity of 98% are given. Applied to a sequential mass testing strategy with a prevalence of 0.5%, there are 328,350 false positive test results (Figure S7). The positive predictive value is 16.74%, which means that 16.74% who test positive are actually positive for SARS-CoV-2. The negative predictive value (NPV) is 99.94%, which means that 99.94% of the individuals who test negative are actually considered negative for SARS-CoV-2.

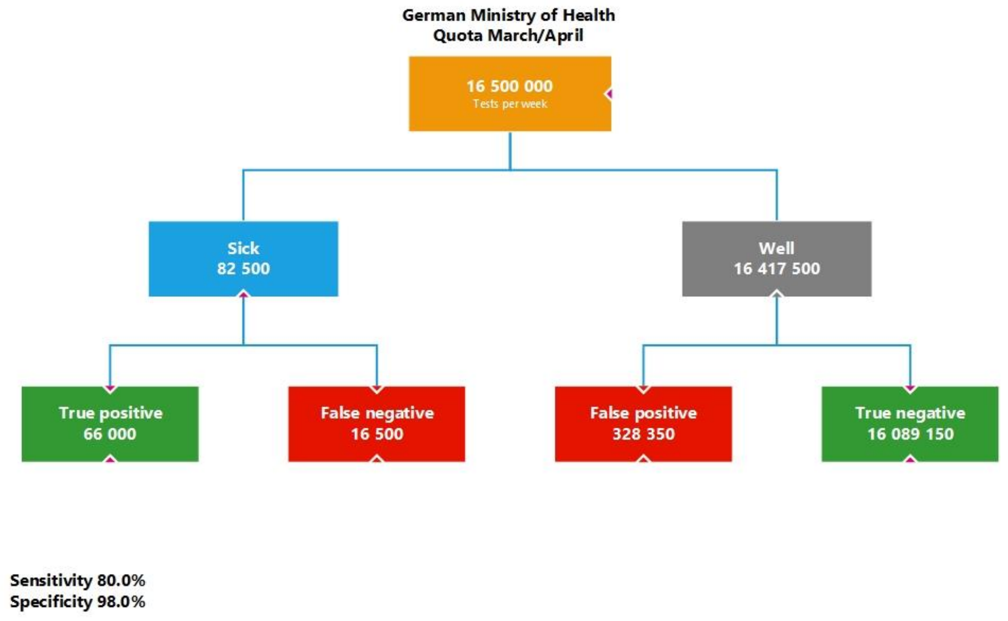

Figure S7. Simulation calculation for the contingent data of the Federal Ministry of Health [18] with sequential testing strategy and prevalence of 0.5%.

If the 394,350 persons identified as positive (66,000 true positives + 328,350 false positives) are now retested with a SARS-CoV-2 PCR test (sensitivity 99.7%, specificity 98.6%), a prevalence of 16.74% is assumed as this is the pre-test probability in the scope of the PPV. Under these conditions there would still be 4597 false positives who would be unjustifiably sent to quarantine. This illustrates that an increasing absolute number of tests results in an increasing absolute number of false positive test results. The additional rapid antigen tests would increase the 7-day incidence by (65,816 + 4597)/831.9 = 84.64/100,000 (Figure S8).

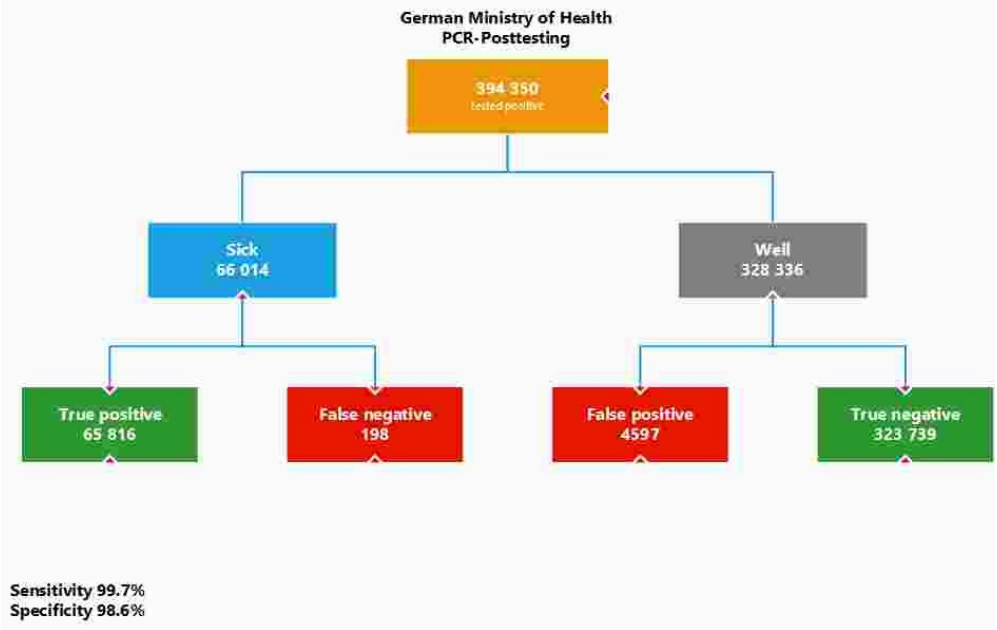

Figure S8. Simulation calculation for the contingent data of the Federal Ministry of Health from Figure S7 with a pre-test probability of 16.74%.

Additional calculations

An earlier review [19] found a sensitivity of 64.8% and a specificity of 98.0%. Applied to a sequential mass testing strategy with a prevalence of 0.5%, there are 19,900 false positive test results (Figure S9). The positive predictive value (PPV) is 14.00%, which means that 14.00% of persons who test positive are actually positive for SARS-CoV-2. The negative predictive value (NPV) is 99.82%, which means that 99.82% of the people who show a negative test result are actually considered negative for SARS-CoV-2. If the 23,140 people identified as positive (3240 true positives + 19,900 false positives) are now retested with a SARS-CoV-2 PCR test (sensitivity 99.7%, specificity 98.6%), a prevalence of 14.00% is assumed, as this is the pre-test probability to the extent of the PPV. Under these assumptions, 279 individuals would still result as false positives who would be wrongly sent to quarantine. The additional rapid antigen tests would increase the 7-day incidence by 4.22/100,000 (Figure S10).

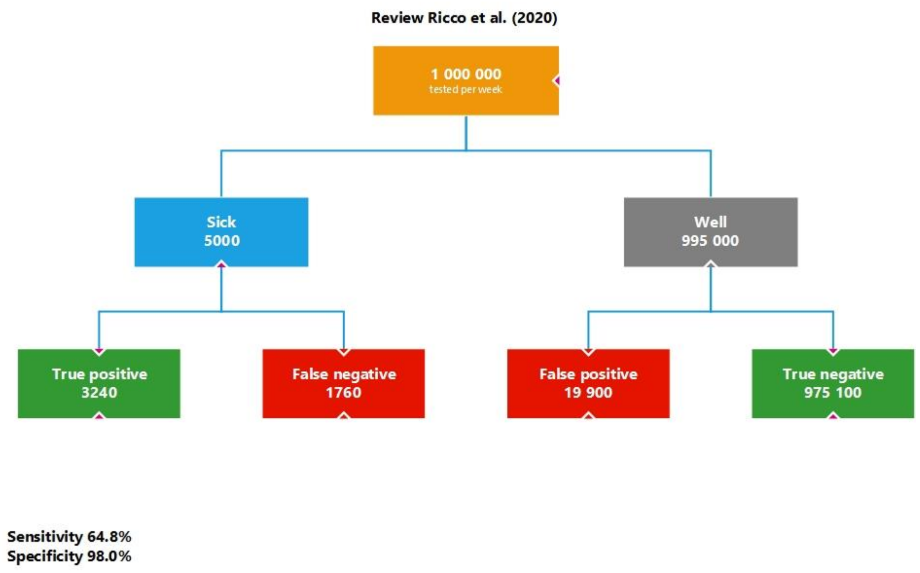

Figure S9. Simulation calculation for average quality criteria in a previous review [19] with sequential testing strategy and prevalence of 0.5%.

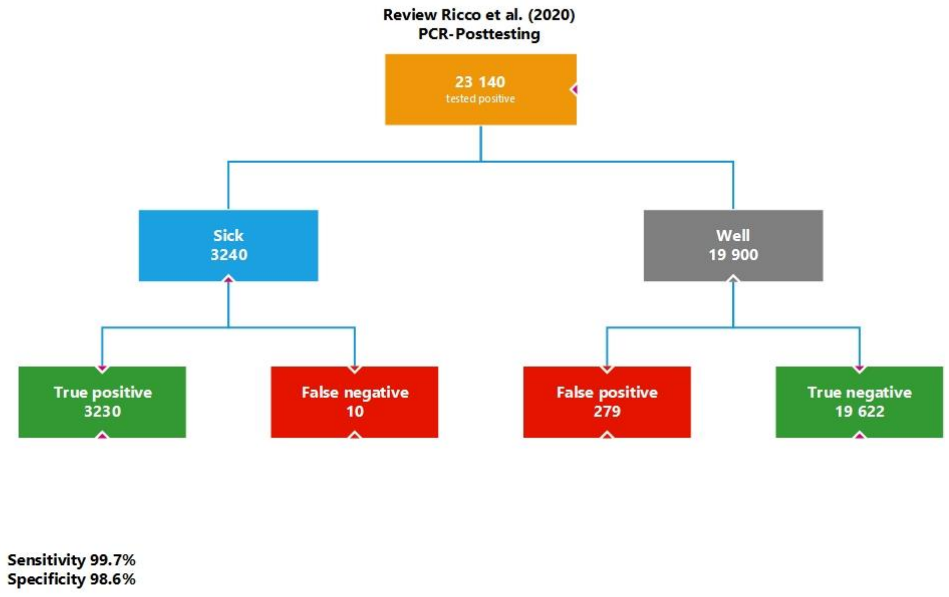

Figure S10. Simulation calculation for PCR post-testing of positive test results from a previous review [19] from Figure S9 with a pre-test probability of 14.00%.

Mass testing was conducted in a public square in San Francisco [10]. According to the original manufacturer's criteria, a sensitivity of 71.4%, and a specificity of 95.7% resulted. Applied to a sequential mass testing strategy with a prevalence of 0.5%, there are 42,785 false positive test results (Figure S11). The positive predictive value is 7.70%, which means that 7.70% of persons who test positive are actually positive for SARS-CoV-2. The negative predictive value (NPV) is 99.85%, which means that 99.85% of the people for whom a negative test result is indicated are actually considered negative for SARS-CoV-2. If the 46,355 people identified as positive (3570 true positives + 42,785 false positives) are now retested with a SARS-CoV-2 PCR test (sensitivity 99.7%, specificity 98.6%), a prevalence of 7.70% is assumed, as this is the pre-test probability at the level of the PPV. Under these

conditions, there would still be 599 false positives who would be wrongly quarantined. The additional rapid antigen tests would increase the 7-day incidence by 5.00/100,000 (Figure S12).

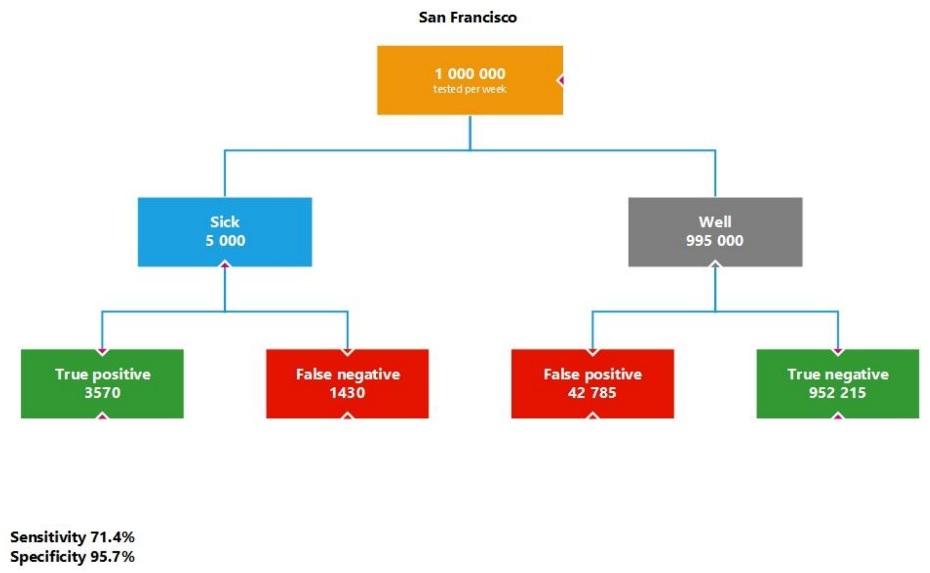

Figure S11. Simulation calculation for the public testing data in San Francisco [10] with sequential testing strategy and prevalence of 0.5%.

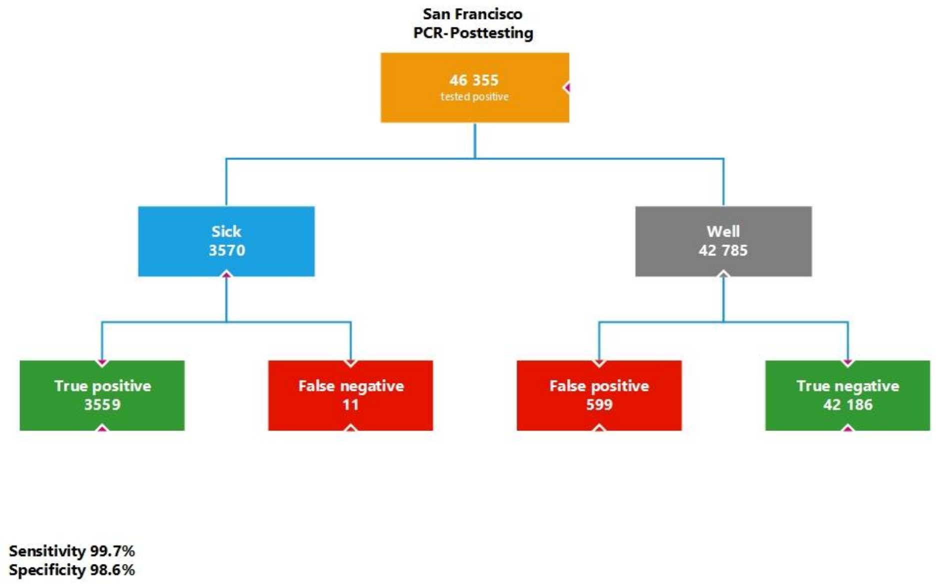

Figure S12. Simulation calculation for PCR post-testing of positive test results with the public testing data in San Francisco [10] from Figure S11 with a pre-test probability of 7.70%.

The Lumipulse® antigen test was recommended by the authors for mass testing [11] and will therefore be examined more closely in simulation calculations. A sensitivity of 100.0% and a specificity of 94.8% were given. Applied to a sequential mass testing strategy with a prevalence of 0.5%, 51,740 false positive test results are obtained (Figure S13). The positive predictive value is 8.81%, which means that 8.81% of persons who test positive are actually positive for SARS-CoV-2. The negative predictive

value (NPV) is 100.0%, which means that 100.0% of the people who show a negative test result are actually considered negative for SARS-CoV-2. If the 56,740 people identified as positive (5000 true positives + 51,740 false positives) are now retested with a SARS-CoV-2 PCR test (sensitivity 99.7%, specificity 98.6%), a prevalence of 8.81% is assumed as this is the pre-test probability to the extent of the PPV. Under these conditions, there would still be 724 false positives who would be wrongly quarantined. The additional rapid antigen tests would increase the 7-day incidence by 6.86/100,000 (Figure S14).

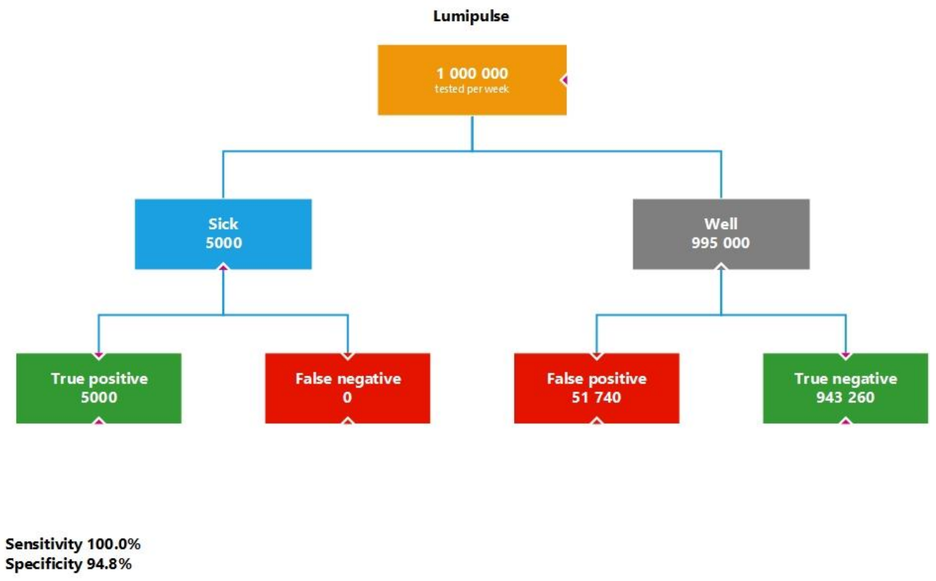

Figure S13. Simulation calculation for the data of the Lumipulse® antigen test [11] with sequential test strategy and prevalence of 0.5%.

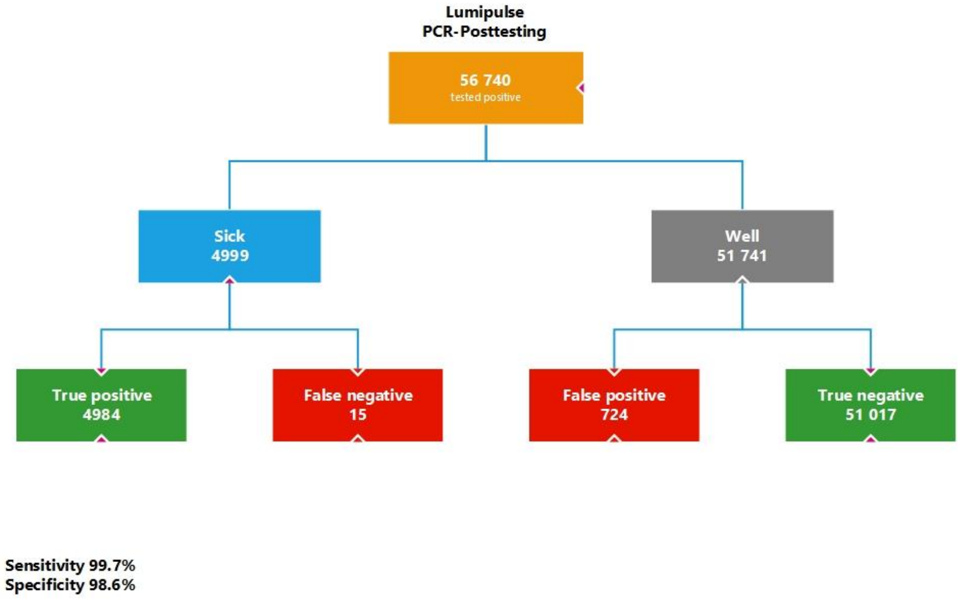

Figure S14. Simulation calculation for the PCR post-testing of the positive test results with the data of the Lumipulse® Antigen Test [11] from Figure S13 with a pre-test probability of 8.81%.

References

1. Lindner AK, Nikolai O, Rohardt C, et al. (2021) Head-to-head comparison of SARS-CoV-2 antigen-detecting rapid test with professional-collected nasal versus nasopharyngeal swab. *Eur Respir J* 57: 2003961.
2. Merino P, Guinea J, Muñoz-Gallego I, et al. (2021) Multicenter evaluation of the Panbio™ COVID-19 rapid antigen-detection test for the diagnosis of SARS-CoV-2 infection. *Clin Microbiol Infect* 27: 758–761.
3. Kohmer N, Toptan T, Pallas C, et al. (2021) The comparative clinical performance of four SARS-CoV-2 rapid antigen tests and their correlation to infectivity in vitro. *J Clin Med* 10: 328.
4. Osterman A, Baldauf HM, Eletreby M, et al. (2021) Evaluation of two rapid antigen tests to detect SARS-CoV-2 in a hospital setting. *Med Microbiol Immunol* 210: 65–72.
5. Cerutti F, Burdino E, Milia MG, et al. (2020) Urgent need of rapid tests for SARS CoV-2 antigen detection: evaluation of the SD-Biosensor antigen test for SARS-CoV-2. *J Clin Virol* 132: 104654.
6. Corman VM, Haage VC, Bleicker T, et al. (2020) Comparison of seven commercial SARS-CoV-2 rapid point-of-care antigen tests: a single-centre laboratory evaluation study. *Lancet Microbe* 2: e311–e319.
7. Krüger LJ, Gaeddert M, Köppel L, et al. (2020) Evaluation of the accuracy, ease of use and limit of detection of novel, rapid, antigen-detecting point-of-care diagnostics for SARS-CoV-2. *medRxiv*.
8. Mina MJ, Peto TE, García-Fiñana M, et al. (2021) Clarifying the evidence on SARS-CoV-2 antigen rapid tests in public health responses to COVID-19. *Lancet* 397: 1425–1427.
9. Scohy A, Anantharajah A, Bodéus M, et al. (2020) Low performance of rapid antigen detection test as frontline testing for COVID-19 diagnosis. *J Clin Virol* 129: 104455.
10. Pilarowski G, Lebel P, Sunshine S, et al. (2020) Performance characteristics of a rapid SARS-CoV-2 antigen detection assay at a public plaza testing site in San Francisco. *J Infect Dis* 223: 1139–1144.
11. Gili A, Paggi R, Russo C, et al. (2021) Evaluation of Lumipulse® G SARS-CoV-2 antigen assay automated test for detecting SARS-CoV-2 nucleocapsid protein (NP) in nasopharyngeal swabs for community and population screening. *Int J Infect Dis* 105: 391–396.
12. Landaas ET, Storm ML, Tollånes MC, et al. (2021) Diagnostic performance of a SARS-CoV-2 rapid antigen test in a large, Norwegian cohort. *J Clin Virol* 137: 104789.
13. Courtellemont L, Guinard J, Guillaume C, et al. (2021) High performance of a novel antigen detection test on nasopharyngeal specimens for diagnosing SARS-CoV-2 infection. *J Med Virol* 93: 3152–3157.
14. Mboumba Bouassa RS, Veyer D, Péré H, et al. (2021) Analytical performances of the point-of-care SIENNA™ COVID-19 Antigen Rapid Test for the detection of SARS-CoV-2 nucleocapsid protein in nasopharyngeal swabs: a prospective evaluation during the COVID-19 second wave in France. *Int J Infect Dis* 106: 8–12.
15. Dinnes J, Deeks JJ, Berhane S, et al. (2021) Rapid, point-of-care antigen and molecular-based tests for diagnosis of SARS-CoV-2 infection. *Cochrane Database Syst Rev* 3: CD013705.
16. Pray IW, Ford L, Cole D, et al. (2021) Performance of an antigen-based test for asymptomatic and symptomatic SARS-CoV-2 testing at two University campuses - Wisconsin, September-October 2020. *MMWR Morb Mortal Wkly Rep* 69: 1642–1647.

17. Robert Koch Institut (RKI). Corona-Schnelltest-Ergebnisse verstehen, Berlin, 2021. Available from: https://web.archive.org/web/20210319165343/https://www.rki.de/DE/Content/InfAZ/N/Neuartiges_Coronavirus/Infografik_Antigentest_PDF.pdf?__blob=publicationFile.
18. Bundesministerium für Gesundheit (German Ministry of Health). Fragen und Antworten zu Schnell- und Selbsttests zum Nachweis von SARS-CoV-2, 2021. Available from: https://web.archive.org/web/20210415083733/https://www.bundesgesundheitsministerium.de/coronavirus/nationale-teststrategie/faq-schnelltests.html.
19. Riccò M, Ferraro P, Gualerzi G, et al. (2020) Point-of-care diagnostic tests for detecting SARS-CoV-2 antibodies: a systematic review and meta-analysis of real-world data. *J Clin Med* 9: 1515.

AIMS Press

© 2022 the Author(s), licensee AIMS Press. This is an open access article distributed under the terms of the Creative Commons Attribution License (http://creativecommons.org/licenses/by/4.0)
