# Supplementary material for: Methodological problems of SARS-CoV-2 rapid point-of-care tests when used in mass testing
Source: AIMS Public Health. 2021 Nov 23;9(1):73–93. doi: 10.3934/publichealth.2022007 (PMC8755956; doi:10.3934/publichealth.2022007)
Supplement: Supplementary file 2 [file publichealth-09-01-007-s002.pdf]

Public Health

http://www.aimspress.com/journal/aimsph

AIMS Public Health, 9(1): 73–93.
DOI: 10.3934/publichealth.2022007
Received: 17 September 2021
Accepted: 16 November 2021
Published: 23 November 2021

Research article

Methodological problems of SARS-CoV-2 rapid point-of-care tests when used in mass testing

Oliver Hirsch[1,*], Werner Bergholz[2], Kai Kisielinski[3], Paul Giboni[4] and Andreas Sönnichsen[5]

[1] Department of Psychology, FOM University of Applied Sciences, Birlenbacher Str. 17, 57078 Siegen, Germany
[2] International Standards Consulting GmbH, 30989 Gehrden, Germany
[3] Private Practice, 40212 Düsseldorf, Germany
[4] Private Practice, 22763 Hamburg, Germany
[5] Department of General Practice and Family Medicine, Center for Public Health, Medical University of Vienna, Wien, Austria

* **Correspondence:** Email: oliver.hirsch@fom.de.

Supplementary Material S2

Additional explanations regarding incidence values

A) Explanation for lower incidence values than predicted:

We have developed a mathematical model which has predicted higher values than are reported at the end of June 2021. The reason for this discrepancy is most likely that several shortcomings of both the actual PCR test procedures and the way the data are processed by the Robert Koch Institute (RKI) could not be incorporated in our model calculations. In detail:

Problem 1:

There is no standardization of the PCR test procedure carried out in the 200 plus laboratories, neither is there a uniform cycle threshold Ct value to render a test positive. This will result in a large variability in the test results, with the most prominent effect on the false positive rate and the positive predictive value PPV. A good illustration of this state of affairs is found in a publication by the RKI in which the set of average positive test value rates per calendar week for each laboratory is visualized

in a boxplot, as shown in Figure S1:

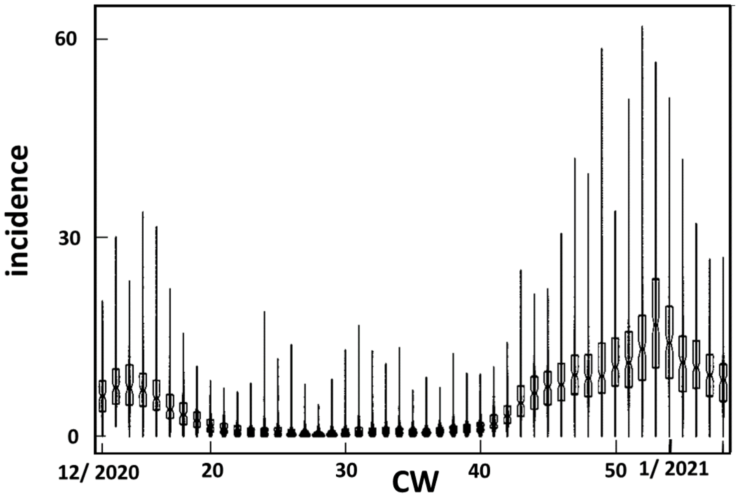

Figure S1. Fraction of SARS-CoV-2 PCR-tests, calendar weeks 12/2020 to 5/2021, own graphical representation, source: Epidemiologisches Bulletin 6/2021, RKI, 11th February 2021.

The most striking feature for all calendar weeks is that the distribution of percentages is asymmetric for all calendar weeks. The asymmetry is the more pronounced the lower the incidence (as e.g. in CW 21 to 41). At the time of developing our mathematical model (March 2021), the incidence has been high, as of June 2021 the situation is similar to CW 21 to 41 in 2020.

Based on this, two conclusions are possible:

1) It appears unlikely that the distribution of positive cases is really asymmetrically distributed. Rather, the most likely reason is the large variance in the tests which are not standardized. This corresponds to industrial production processes with a large process variance which also typically produce asymmetric distributions due to small defects, whereas stable processes tend to result in narrower normal distributions.
2) The degree of asymmetry influences the mathematical model in a significant way, since the averages are dependent on the asymmetry. This was not accounted for in our model since the data supplied by the authorities are not sufficient to do this.

Therefore, we argue that the simplification of the model using averages instead of full distributions has led to the too high prediction of the minimum attainable incidence number.
Another possible reason is the simplification of using the average values for Germany rather than the values for the 16 federal states. Figure S2 shows the striking fact that at a time for which the incidence is low there is a high systematic spread of the incidence:

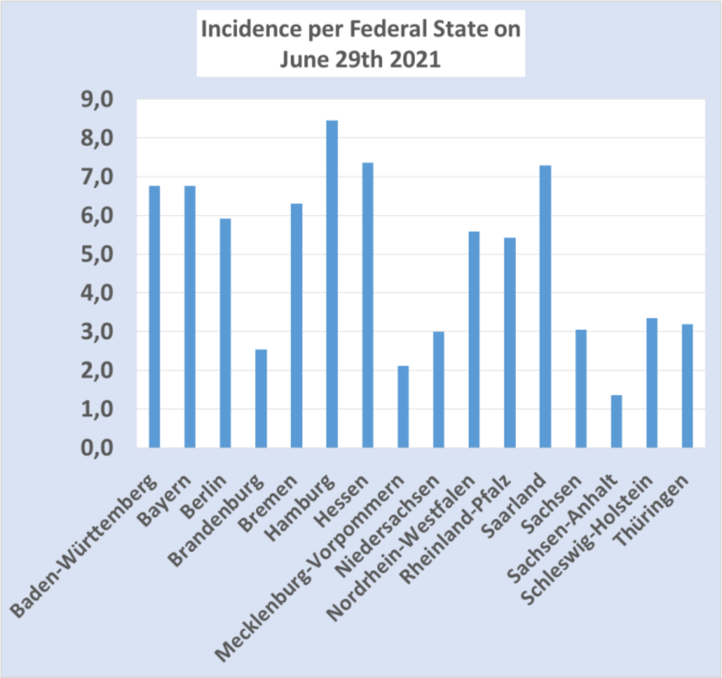

Figure S2. Incidence per federal state on June 29th, 2021, own graphical representation, Source: Daily situation report RKI of June 25th, 2021.

At the end of June there is a factor of approximately 5 scatter of the incidences among the 16 federal states. Therefore, the geographic variation of the measured incidence is another factor which can have an impact on the prediction of the minimum possible incidence.

Problem 2:

The model calculations refer in each case to additional representative population samples that would have to be drawn if the results were comparable. This would be the correct epidemiological procedure. However, the testing strategy behind the test results is unclear. Due to government regulations, massive testing had to take place in settings where low prevalence was expected (schools, workplace, etc.). Testing in environments with lower prevalence of the disease is expected to have a smaller additional impact on the 7-day incidence (see Table S1). However, this does not weaken our conclusions, which refer to the standardized procedure of testing representative population samples from a population with a defined prevalence. One could argue that the government's testing strategy would then probably underestimate the actual 7-day incidence. Here, however, the discussion arises again with regard to the clinical significance of a representative 7-day incidence, which is to a considerable extent based on a SARS-CoV-2 PCR test with Ct values that are clearly too high, so that this parameter is not able to reflect the actual incidence of infection.

Table S1. Additional increases in 7-day incidence per 100,000 depends on different prevalences of COVID-19. Maximum calculations using the contingent data for self-tests from the German Federal Ministry of Health are performed using 16,500,000 tests per week and sequential SARS-CoV-2 PCR testing.

Prevalence	Increase in 7-day incidence per 100,000
0.4%	68.8
0.3%	53.0
0.25%	45.1
0.2%	37.2
0.1%	21.4
0.05%	13.5

B) Positive Proof of an incidence increase by introduction of POC rapid antigen tests:

Although the accuracy of the mathematical model presented is not as high as expected (for the reasons explained above), the qualitative prediction is correct as shown by recent data published by the RKI. These constitute proof that as predicted the introduction of rapid antigen tests as documented in the weekly reports of the RKI lead to an increase of positive PCR tests. Figure S3 taken from the weekly report dated June 1st, 2021, shows a clear increase in the number of positive PCR tests at the onset of rapid antigen tests at schools:

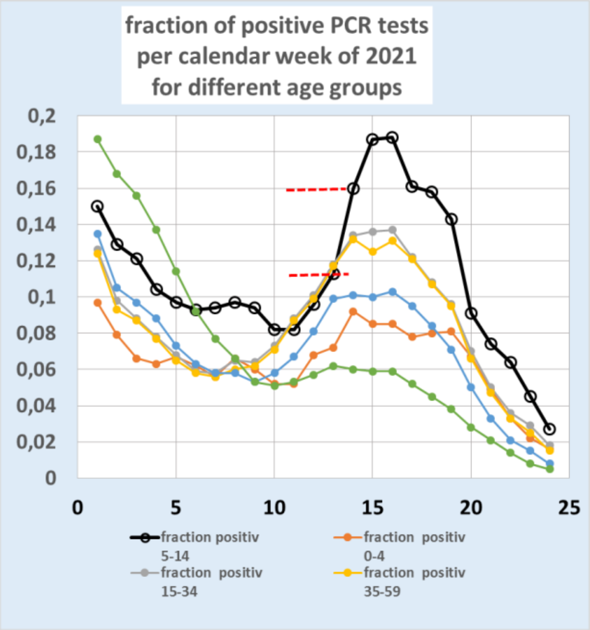

Figure S3. Percentage of positive PCR tests for different age groups as a function of calendar week. Own graphical representation, source: Laboratory-based surveillance of SARS-CoV-2, weekly report of 01.06.2021.

It is clear that as of calendar week 14 the percentage of positive tests for the age group 5 to 14 jumps from about 12% to approximately 19% and has remained higher than the values for all other cohorts, which are not subject to regular rapid antigen tests.

AIMS Press

© 2022 the Author(s), licensee AIMS Press. This is an open access article distributed under the terms of the Creative Commons Attribution License (http://creativecommons.org/licenses/by/4.0)
